# EPR-Net: constructing a non-equilibrium potential landscape via a variational force projection formulation

**DOI:** 10.1093/nsr/nwae052

**Published:** 2024-02-20

**Authors:** Yue Zhao, Wei Zhang, Tiejun Li

**Affiliations:** Center for Data Science, Peking University, Beijing 100871, China; Zuse Institute Berlin, Berlin 14195, Germany; Department of Mathematics and Computer Science, Freie Universität Berlin, Berlin 14195, Germany; Center for Data Science, Peking University, Beijing 100871, China; Laboratory of Mathematics and Applied Mathematics (LMAM) and School of Mathematical Sciences, Peking University, Beijing 100871, China; Center for Machine Learning Research, Peking University, Beijing 100871, China

**Keywords:** high-dimensional potential landscape, non-equilibrium system, entropy production rate, dimensionality reduction, deep learning

## Abstract

We present EPR-Net, a novel and effective deep learning approach that tackles a crucial challenge in biophysics: constructing potential landscapes for high-dimensional non-equilibrium steady-state systems. EPR-Net leverages a nice mathematical fact that the desired negative potential gradient is simply the orthogonal projection of the driving force of the underlying dynamics in a weighted inner-product space. Remarkably, our loss function has an intimate connection with the steady entropy production rate (EPR), enabling simultaneous landscape construction and EPR estimation. We introduce an enhanced learning strategy for systems with small noise, and extend our framework to include dimensionality reduction and the state-dependent diffusion coefficient case in a unified fashion. Comparative evaluations on benchmark problems demonstrate the superior accuracy, effectiveness and robustness of EPR-Net compared to existing methods. We apply our approach to challenging biophysical problems, such as an eight-dimensional (8D) limit cycle and a 52D multi-stability problem, which provide accurate solutions and interesting insights on constructed landscapes. With its versatility and power, EPR-Net offers a promising solution for diverse landscape construction problems in biophysics.

## Teaser

EPR-Net: Quantifying the Waddington landscape for non-equilibrium steady-state systems in high dimensions via elegant variational formulations with statistical physics interpretation.

## INTRODUCTION

Since Waddington’s famous landscape metaphor on the development of cells in the 1950s [1], the construction of potential landscapes for non-equilibrium biochemical reaction systems has been recognized as an important problem in theoretical biology, as it provides insightful pictures for understanding complex dynamical mechanisms of biological processes. This problem has attracted considerable attention in recent decades in both biophysics and applied mathematics communities. Until now, different approaches have been proposed to realize Waddington’s landscape metaphor in a rational way, and its connection to computing the optimal epigenetic switching paths and switching rates in biochemical reaction systems has also been extensively studied. See [2–13] and the references therein for details and [14–18] for reviews. Broadly speaking, these proposals can be classified into two types: (T1) the construction of a potential landscape in the finite noise regime [2–5] and (T2) the construction of the quasi-potential in the zero-noise limit [6–8].

For low-dimensional systems (i.e. dimension less than 4), the potential landscape can be numerically computed either by solving a Fokker–Planck equation (FPE) using grid-based methods until the steady solution is reached approximately as in (T1)-type proposals [3,19], or by solving a Hamilton–Jacobi–Bellman (HJB) equation using, for instance, the ordered upwind method [20] or minimum action–type method [8] as in (T2)-type proposals. However, these approaches suffer from the curse of dimensionality when applied to high-dimensional systems. Although methods based on mean-field approximations are able to provide a semi-quantitative description of the potential landscape for some typical systems [4,21], direct and general approaches are still favored in applications. In this aspect, pioneering work has been done recently, which allows direct construction of a high-dimensional potential landscape using deep neural networks (DNNs), based on either the steady viscous HJB equation satisfied by the potential landscape function in the (T1) case [22,23], or the *pointwise* orthogonal decomposition of the force field in the (T2) case [24].

While these works have significantly advanced methodological developments, these approaches are based on solving HJB equations alone and may encounter numerical difficulties due to either non-uniqueness of the weak solution to the non-viscous HJB equation in the (T2) case [25], or singularity of the solution with small noise in the (T1) case. Meanwhile, the loss functions considered in [22–24] are essentially of physics-informed neural network (PINN) form [26], so are generally non-convex, and thus might encounter troubling local minimum issues in the training process.

In this work, we present a simple yet efficient DNN approach to construct the potential landscape of high-dimensional non-equilibrium steady-state (NESS) systems of (T1) type with either moderate or small noise. Its intimate connection with non-equilibrium statistical mechanics, nice variational structure and superior numerical performance make it a competitive and promising approach in landscape construction methodology. Our main contributions are as follows.

(1) *Proposal of entropy production rate (EPR) loss.* We introduce the convex EPR loss function, reveal its connection with the entropy production rate in statistical physics and propose its enhanced version using the tempering technique.
(2) *Dimensionality reduction.* We put forward a simple dimensionality reduction strategy when the reducing variables are prescribed, and, interestingly, the reduction formalism has a unified formulation with the EPR framework for the primitive variables. This even holds whenever the system has constant or variable diffusion coefficients.
(3) *Successful high-dimensional applications.* We successfully apply our approach to some challenging high-dimensional biological systems, including an eight-dimensional (8D) cell cycle model [4] and a 52D multi-stable system [27]. Our results reveal more delicate structure of the constructed landscapes than what mean-field approximations typically provide, yet we acknowledge that mean-field approximations are not limited by the potential challenges in stochastic differential equation simulations.

Overall, EPR-Net offers a promising solution for diverse landscape construction problems in biophysics. Even its nice mathematical structure and connection with non-equilibrium statistical physics make it a unique object that deserves further theoretical and numerical exploration in the future.

## EPR FRAMEWORK

In this section, we provide an overview of the whole EPR framework, including its primitive formulation, the physical interpretation, its convex property and the enhanced EPR version.

### Overview

Consider an ergodic stochastic differential equation (SDE)


(1) \begin{eqnarray*}
\frac{{\rm d} \boldsymbol{x}(t)}{{\rm d} t}=\boldsymbol{F}(\boldsymbol{x}(t))+\sqrt{2D}\, \dot{\boldsymbol{w}},\qquad \boldsymbol{x}(0)=\boldsymbol{x}_0,
\end{eqnarray*}

where $\boldsymbol{x}_0\in \mathbb {R}^d$, $\boldsymbol{F}:\mathbb {R}^d\rightarrow \mathbb {R}^d$ is a smooth driving force, $\dot{\boldsymbol{w}}=(\dot{w}_1,\ldots ,\dot{w}_d)^\top$ is the *d*-dimensional temporal Gaussian white noise with $\mathbb {E}\dot{w}_i(t)=0$ and $\mathbb {E}[\dot{w}_i(t)\dot{w}_j(s)]=\delta _{ij}\delta (t-s)$ for *i, j* = 1, …, *d* and *s, t* ≥ 0. The constant *D* > 0 specifies the noise strength, which is often proportional to the system’s temperature *T*. We denote by $p_{\mathrm{ss}}(\boldsymbol{x})$ its steady-state probability density function (PDF).

Following the (T1)-type proposal in [3], we define the potential of (1) as *U* = −*D* ln *p*_ss_ and the steady probability flux $\boldsymbol{J}_{\mathrm{ss}}=p_{\mathrm{ss}}\boldsymbol{F} - D \nabla p_{\mathrm{ss}}$ in the domain Ω, which we assume for simplicity is either $\mathbb {R}^d$ or a *d*-dimensional hyperrectangle. The steady-state PDF $p_{\mathrm{ss}}(\boldsymbol{x})$ satisfies the FPE


(2) \begin{eqnarray*}
-\nabla \cdot (p_{\mathrm{ss}}\boldsymbol{F} ) + D \Delta p_{\mathrm{ss}}=0\quad \textrm {in}\,\, \Omega ,
\end{eqnarray*}

and we assume the asymptotic boundary condition (BC) $p_{\mathrm{ss}}(\boldsymbol{x})\rightarrow 0$ as $|\boldsymbol{x}|\rightarrow \infty$ when $\Omega =\mathbb {R}^d$, or the reflecting boundary condition $\boldsymbol{J}_{\mathrm{ss}}\cdot \boldsymbol{n}=0$ when $\Omega \subset \mathbb {R}^d$ is a *d*-dimensional hyperrectangle, where $\boldsymbol{n}$ is the unit outer normal. In both cases, we have $p_{\mathrm{ss}}(\boldsymbol{x})\ge 0$ and $\int _{\Omega } p_{\mathrm{ss}}(\boldsymbol{x})\, {\rm d} \boldsymbol{x}=1$.

**Figure 1.**
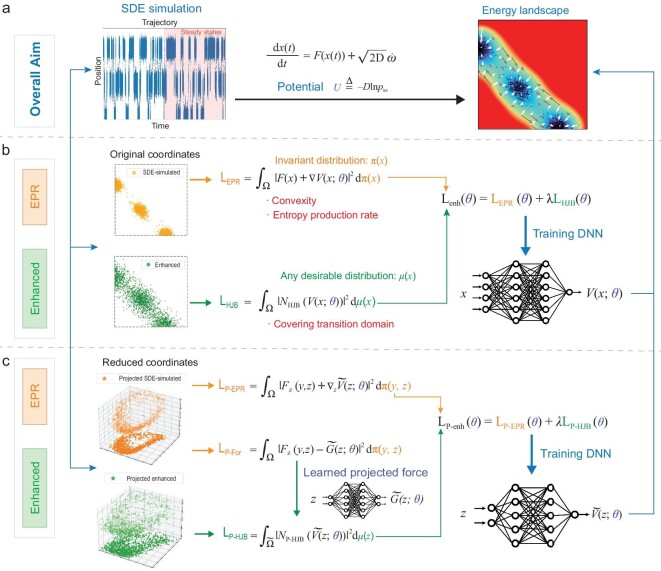
Constructing energy landscapes through enhanced EPR workflow. (a) The primary objective is to construct the energy landscape defined through the steady-state distribution of the system. (b) Constructing the high-dimensional energy landscape using the EPR framework with primitive variables. (c) Constructing the dimensionality-reduced energy landscape using EPR with prescribed reduced variables.

Here we illustrate the main EPR workflow through Fig. 1. As depicted in Fig. 1a, our primary objective is to construct the energy landscape *U* = −*D* ln *p*_ss_ defined for Equation (1). Leveraging our proposed approach, the enhanced EPR method, we first simulate the SDE of interest until steady state is reached in order to get sample points (the whole ‘SDE simulation’ column of Fig. 1). We then train a neural network, representing the potential *U*, for the landscape construction, even when confronted with challenging high-dimensional scenarios. We initially introduce the EPR loss $\mathrm{L_{EPR}}$ (the middle column of Fig. 1b), which benefits from its convexity, with its minimum coinciding with the entropy production rate. Subsequently, we present the enhanced EPR loss $\mathrm{L_{enh}}$, tailored to encompass the transition domain more effectively. Furthermore, the overall methodology can be easily extended to the dimensionality reduction problem (Fig. 1c), with a unified formulation as the EPR framework shown in Fig. 1b, but for the projected variables and corresponding loss functions $\mathrm{L_{P{\rm -}EPR}}$ and $\mathrm{L_{P{\rm -}enh}}$.

### EPR loss

Aiming at an effective approach for high-dimensional applications, we employ NNs to approximate $U(\boldsymbol{x})$, and the key idea in this paper is to learn *U* by training the DNN with the loss function


(3) \begin{eqnarray*}
{\rm L_{EPR}}(V)=\int _{\Omega }|\boldsymbol{F}(\boldsymbol{x})+\nabla {\it V}(\boldsymbol{x};\theta )|^2 \, {\rm d} \pi (\boldsymbol{x}),
\end{eqnarray*}

where $V:=V(\boldsymbol{x};\theta )$ is a neural network function with parameters θ [28], and ${\rm d} \pi (\boldsymbol{x})=p_{\mathrm{ss}}(\boldsymbol{x})\, {\rm d} \boldsymbol{x}$. To justify (3), we note that, for any function *W, U* satisfies the orthogonality relation


(4) \begin{eqnarray*}
\int _{\Omega } (\boldsymbol{F}(\boldsymbol{x})+\nabla U(\boldsymbol{x}))\cdot \nabla W(\boldsymbol{x})\, {\rm d} \pi (\boldsymbol{x}) = 0,
\end{eqnarray*}

which follows from FPE (2), a simple integration by parts and the corresponding BC. Equation (4) means that $\boldsymbol{F}+\nabla U$ is perpendicular to the space $\mathcal {G}$ formed by ∇*W* for all possible *W* under the π-weighted inner product. Equivalently, −∇*U* is the orthogonal projection of the driving force $\boldsymbol{F}$ onto $\mathcal {G}$ under the π-weighted inner product, which implies that *U* is the unique minimizer (up to a constant) of the loss function (3). See Supplementary Section A for detailed derivations. We note that related ideas are taken in coarse graining of molecular systems [29,30] and generative modeling in machine learning [31,32]. However, to the best of our knowledge, using the loss in (3) to construct the potential of NESS systems has never been considered before.

In fact, defining the π-weighted inner product for any square-integrable functions *f, g* on Ω as


(5) \begin{eqnarray*}
(f,g)_\pi :=\int _\Omega f(\boldsymbol{x})g(\boldsymbol{x})\, {\rm d} \pi (\boldsymbol{x}),
\end{eqnarray*}

and the corresponding $L^2_\pi$ norm ‖ · ‖_π_ by $\Vert f\Vert ^2_{\pi }:= (f,f)_\pi$, we get a Hilbert space $L^2_\pi$ (see, e.g. Chapter II.1 of [33]). Equation (4) implies that the minimization of EPR loss gives the orthogonal projection of $\boldsymbol{F}$ to the function gradient space $\mathcal {G}$ which is formed by function gradients ∇*W* for any *W*, under the π-weighted inner product. Choosing *W* = *U* in (4) gives


(6) \begin{eqnarray*}
\boldsymbol{F}(\boldsymbol{x}) &=& {-}\nabla U(\boldsymbol{x})+ \boldsymbol{l}(\boldsymbol{x})\quad \text{such that}\\
&& (\nabla U,\boldsymbol{l})_\pi = 0.
\end{eqnarray*}

However, we remark that this orthogonality holds only in the $L^2_\pi$-inner product sense instead of the pointwise sense. Furthermore, the two orthogonality relations (4) and (6) can be understood as follows. Using (6), relation (4) is equivalent to $\int _{\Omega } \boldsymbol{l} \cdot \nabla W d\pi = 0$ for any *W*. Integration by parts gives $\nabla \cdot (\boldsymbol{l}\, \mathrm{e}^{-U/D})=0$, which is equivalent to $\nabla U \cdot \boldsymbol{l}+ D \nabla \cdot \boldsymbol{l} = 0$. When *D* → 0, we recover the pointwise orthogonality, which is adopted in computing quasi-potentials in [24]. In mathematical language (6) can be understood as the Hodge-type decomposition in $L^2_\pi$ instead of *L*^2^ space.

To utilize (3) in numerical computations, we replace the ensemble average (3) with respect to the unknown π by the empirical average with data sampled from (1):


(7) \begin{eqnarray*}
{\rm \widehat{L}_{EPR}}(\theta )=\frac{1}{N}\sum _{i=1}^N |\boldsymbol{F}(\boldsymbol{x}_i)+\nabla V(\boldsymbol{x}_i;\theta )|^2.
\end{eqnarray*}

Here $(\boldsymbol{x}_i)_{1\le i \le N}$ could be either the final states (at time $\rm {T}$) of *N* independent trajectories starting from different initializations, or equally spaced time series along a single long trajectory up to time $\rm {T}$, where $\rm {T} \gg 1$. In both cases, the ergodicity of SDE in (1) guarantees that (7) is a good approximation of (3) as long as $\rm {T}$ is large [34]. We adopt the former approach in the numerical experiments in this work where the gradients of both *V* (with respect to $\boldsymbol{x}$) and the loss itself (with respect to θ) in (7) are calculated by auto-differentiation through PyTorch [35]. Given the involvement of an approximation on π we perform a formal stability analysis of (3) in Supplementary Section B to ensure its reliability and robustness. Additionally in the following subsection, we elucidate the benefits of EPR, particularly in terms of *convexity* and its *physical interpretation* pertaining to the entropy production rate.

### Physical interpretation and convexity

The minimum loss of (3), denoted ${\rm L_{EPR}}(U)$, possesses a well-defined physical interpretation. In the following discussion, we show that this minimum EPR loss aligns precisely with the steady entropy production rate as defined in NESS theory. Following [36,37] we have the following important identity concerning the entropy production for (1):


(8) \begin{eqnarray*}
D\frac{{\rm d} S(t)}{{\rm d} t} = e_p(t)-h_d(t).
\end{eqnarray*}

Here $S(t):=-\int _\Omega p(\boldsymbol{x},t)\ln p(\boldsymbol{x},t)\, {\rm d} \boldsymbol{x}$ is the entropy of the probability density function $p(\boldsymbol{x},t)$ at time *t, e_p_* is the EPR


(9) \begin{eqnarray*}
e_p(t) = \int _\Omega |\boldsymbol{F} - D \nabla \ln p |^2 p(\boldsymbol{x},t)\, {\rm d} \boldsymbol{x},
\end{eqnarray*}

and *h_d_* is the heat dissipation rate


(10) \begin{eqnarray*}
h_d(t) = \int _\Omega \boldsymbol{F}(\boldsymbol{x})\cdot \boldsymbol{J}(\boldsymbol{x},t) \, {\rm d} \boldsymbol{x}
\end{eqnarray*}

with the probability flux $\boldsymbol{J}(\boldsymbol{x},t) := p(\boldsymbol{x},t)(\boldsymbol{F}(\boldsymbol{x}) - D\nabla \ln p(\boldsymbol{x},t))$ at time *t*. When *D* = *k_B_T*, the above formulae have clear physical meanings in statistical physics.

From the loss in (3) and the steady state of (9), we get the identity


(11) \begin{eqnarray*}
{\rm L_{EPR}}(U)&=&\int _{\Omega } |\boldsymbol{J}_{\mathrm{ss}}|^2\frac{1}{p_{\mathrm{ss}}}\, {\rm d} \boldsymbol{x} \\
& =&\int _\Omega |\boldsymbol{F} - D \nabla \ln p_{\mathrm{ss}} |^2 p_\mathrm{ss}\, {\rm d} \boldsymbol{x} \\
&=&e^{\rm {ss}}_p,
\end{eqnarray*}

where $\boldsymbol{J}_{\mathrm{ss}}(\boldsymbol{x})$ is the steady probability flux and $e^{\rm {ss}}_p$ denotes the steady EPR of the NESS system (1) [3,36,37]. Therefore, minimizing (3) is equivalent to approximating the steady EPR. This correspondence provides a rationale for naming our approach ‘EPR-Net’.

EPR loss has an appealing property that it is strictly convex on *V* (up to a constant), i.e.


(12) \begin{eqnarray*}
{\rm L_{EPR}}(V_\omega )\< (1-\omega ){\rm L_{EPR}}(V_0)+\omega {\rm L_{EPR}}(V_1)
\end{eqnarray*}

for any 0 < ω < 1 and *V*_0_, *V*_1_ that satisfy $\nabla (V_0-V_1)\not\equiv 0$, where *V*_ω_ ≔ (1 − ω)*V*_0_ + ω*V*_1_. Equation (12) can be easily verified by direct calculations. This strict convexity guarantees the uniqueness of the critical point *V* (up to a constant) and provides a theoretical guarantee on the fast convergence of the training procedure under certain assumptions [38]. It may also contribute to better convergence behavior of EPR during the training process as mentioned in the subsection entitled ‘Numerical comparisons’ below.

### Enhanced EPR loss

Substituting the relation $p_{\mathrm{ss}}(\boldsymbol{x})= \exp (-U(\boldsymbol{x})/D)$ into (2), we get the viscous HJB equation


(13) \begin{eqnarray*}
\mathcal {N}_{\textrm {HJB}}(U)&:=& -\boldsymbol{F} \cdot \nabla U+ D \Delta U- |\nabla U|^2\\
&& +\, D \nabla \cdot \boldsymbol{F} =0
\end{eqnarray*}

with the asymptotic BC *U* → ∞ as $|\boldsymbol{x}|\rightarrow \infty$ in the case of $\Omega =\mathbb {R}^d$, or the reflecting BC $(\boldsymbol{F}+\nabla U) \cdot \boldsymbol{n}=0$ on ∂Ω when Ω is a *d*-dimensional hyperrectangle. As in the framework of PINNs [26] (13) motivates the HJB loss


(14) \begin{eqnarray*}
{\rm L_{HJB}}(V)= \int _{\Omega }|\mathcal {N}_{\textrm {HJB}}(V(\boldsymbol{x};\theta ))|^2 \, {\rm d} \mu (\boldsymbol{x}),
\end{eqnarray*}

where μ is any desirable distribution. By choosing μ properly, this loss allows the use of sample data that better covers the domain Ω and, when combined with the loss in (3), leads to improvement of the training results when *D* is small. Specifically, for small *D*, we propose the enhanced loss that has the form


(15) \begin{eqnarray*}
\mathrm{\widehat{L}_{enh}}(\theta )=\mathrm{\widehat{L}_{EPR}}(\theta )+\lambda \mathrm{\widehat{L}_{HJB}}(\theta ),
\end{eqnarray*}

where ${\rm \widehat{L}_{EPR}}(\theta )$ is defined in (7) and ${\rm \widehat{L}_{HJB}}(\theta ) = ({1}/{N^{\prime }})\sum _{i=1}^{N^{\prime }} |\mathcal {N}_{\textrm {HJB}}(V(\boldsymbol{x}^{\prime }_i;\theta ))|^2$ is an approximation of (14) using an independent data set $(\boldsymbol{x}^{\prime }_i)_{1\le i\le N^{\prime }}$ obtained by sampling the trajectories of (1) with a larger *D*′ > *D*. The weight parameter λ > 0 balances the contribution of the two terms in (15). While its value can be tuned based on the system’s properties, in our numerical experiment we observed that the method performs well for λ taking values in a relatively broad range. Note that the proposed strategy is both general and easily adaptable. For instance, one can alternatively use data $(\boldsymbol{x}^{\prime }_i)_{1\le i\le N^{\prime }}$ that contain more samples in the transition region, or employ in (15) a modification of loss (14) [22]. We further illustrate the motivation of enhanced EPR in Supplementary Section E.

## RESULTS FOR ENERGY LANDSCAPE CONSTRUCTION

In this section we demonstrate the superiority of enhanced EPR over HJB loss alone and over the normalizing flow (NF), which is a class of generative models used for density estimation that leverage invertible transformations to map between complex data distributions and simple latent distributions [39] through 2D benchmark examples. We then apply enhanced EPR to the 3D Lorenz model and a 12D Gaussian mixture model to show its effectiveness in constructing energy landscapes in higher dimensions. We remark that alternative approaches have been investigated for potential construction in limit cycles [40] and the Lorenz system [41] distinct from our methodology.

### Two-dimensional benchmark examples

Within this subsection, we undertake a comparative analysis of 2D benchmark problems. These encompass a toy model, a 2D biological system exhibiting a limit cycle [3] and a 2D multi-stable system [5]; see Supplementary Sections F.1–F.4 for training details, additional results and problem settings.

#### Constructing landscapes

The potential function $V(\boldsymbol{x}; \theta )$ is approximated using a feedforward neural network architecture consisting of three hidden layers, employing the tanh activation function. Each hidden layer comprises 20 hidden states. Subsequently, we refer to the enhanced loss as


(16) \begin{eqnarray*}
\mathrm{L_{enh}} = \lambda _1 \mathrm{L_{EPR}} + \lambda _2 \mathrm{L_{HJB}},
\end{eqnarray*}

where λ_1_ and λ_2_ should be chosen to balance the corresponding loss terms. In Supplementary Section F.1, we conduct a sensitivity analysis of λ_1_, demonstrating that our methods are robust and effective across a broad spectrum of scenarios.

**Figure 2.**
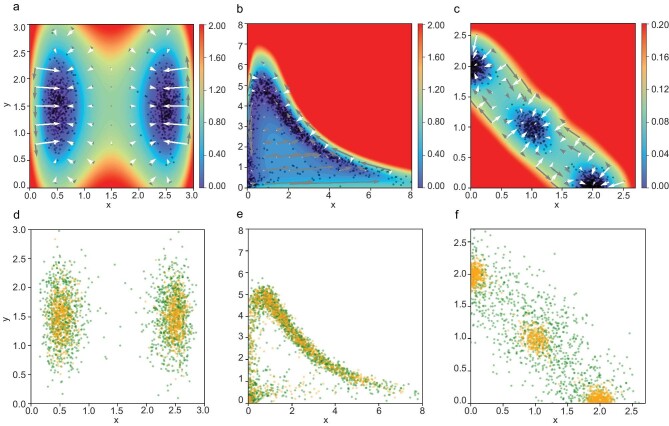
Two-dimensional benchmark examples solved under the EPR framework. (a–c) Filled contour plots of the learned potential $V(\boldsymbol{x};\theta ^{*})$ for (a) a toy model learned by the EPR loss (3), (b) a biochemical oscillation network model [3] and (c) a tri-stable cell development model [5], all of which are learned by the enhanced loss (15). The force field $\boldsymbol{F}(\boldsymbol{x})$ is decomposed into the gradient part $-\nabla V(\boldsymbol{x};\theta ^{*})$ (white arrows) and the non-gradient part $\boldsymbol{F}(\boldsymbol{x})+\nabla V(\boldsymbol{x};\theta ^{*})$ (gray arrows). The length of an arrow corresponds to the magnitude of the vector. The solid dots are samples from the simulated invariant distribution. (d–f) SDE-simulated samples $(\boldsymbol{x}_i)_{1\le i\le N}$ (yellow points) and enhanced samples $(\boldsymbol{x}^{\prime }_i)_{1\le i\le N^{\prime }}$ (green points): (d) *D* = 0.1, where enhanced samples are generated with *D* ′ = 2*D*; (e) *D* = 0.1, where enhanced samples are obtained by adding Gaussian perturbations with σ = 0.05 on SDE-simulated samples; (f) *D* = 0.01, where enhanced samples are generated with *D* ′ = 10*D*.

As illustrated in Fig. 2a–c, we initially showcase well-constructed landscapes under the EPR framework. These include the acquired potentials $V(\boldsymbol{x};\theta ^{*})$, the decomposition of forces and sample points derived from the simulated invariant distribution. In the toy model (Fig. 2a), the gradient of the potential (white arrows) points directly towards the corresponding attractor, while the non-gradient part of the force field (gray arrows) shows a counter-clockwise rotation in the model with a limit cycle (Fig. 2b), and a splitting-and-back flow from the attractor in the middle to the other two attractors in the tri-stable dynamical model (Fig. 2c).

Data enhancement techniques employed to generate improved samples are flexible, as visually depicted in Fig. 2(d–f). We present choices for obtaining these samples, including generating more diffusive samples with a diffusion coefficient *D*′ > *D* or introducing Gaussian perturbations with a standard deviation of σ. We provide a more comprehensive analysis of the data enhancement in Supplementary Section F.1, demonstrating the resilience of enhanced EPR to variations in the enhanced samples. Specifically, in Fig. 2, we use *D*′ = 2*D* for the toy model, *D*′ = 10*D* for the multi-stable problem and σ = 0.05 for the limit-cycle problem. We use the same size SDE-simulated dataset $(\boldsymbol{x}_i)_{1\le i\le N}$ and enhanced dataset $(\boldsymbol{x}^{\prime }_i)_{1\le i\le N^{\prime }}$, denoted *N* = *N*′.

#### Numerical comparisons

We proceed to perform a quantitative and comprehensive comparison of the performance between enhanced EPR, solving HJB alone and NF. For the toy model, the true solution is analytically known (see Supplementary Section F.2), while for the other two 2D examples, we compute the reference solution by discretizing the steady FPE using a piecewise bilinear finite-element method on a fine rectangular grid and computing the obtained sparse linear system using the least-squares solver (the normalization condition $\int _{\Omega } p_{\rm {ss}}(\boldsymbol{x}){\rm d} \boldsymbol{x}=1$ is utilized to fix the additive constant). After training, we shift the minimum of the potentials to the origin and we measure their accuracy using the relative root-mean-square error (rRMSE) and the relative mean absolute error (rMAE),


(17) \begin{eqnarray*}
{\rm rRMSE} =\sqrt{\frac{\int _{\mathcal {D}}\left|V(\boldsymbol{x};\theta ^{*})-U_0(\boldsymbol{x})\right|^2 {\rm d} \boldsymbol{x}}{\int _{\mathcal {D}}|U_0(\boldsymbol{x})|^2 {\rm d} \boldsymbol{x}}},
\end{eqnarray*}

(18) \begin{eqnarray*}
{\rm rMAE} =\frac{\int _{\mathcal {D}}\left|V(\boldsymbol{x};\theta ^{*})-U_0(\boldsymbol{x})\right| {\rm d} \boldsymbol{x}}{\int _{\mathcal {D}}|U_0(\boldsymbol{x})| {\rm d} \boldsymbol{x}},
\end{eqnarray*}

on the domain $\mathcal {D} = \lbrace \boldsymbol{x} \in \Omega | U_0(\boldsymbol{x}) \le 20 D\rbrace$, where *U*_0_ denotes the reference solution.

We present a detailed comparison of the 2D problems in Table 1. Our experiments involve solving HJB loss alone with various distributions of enhanced samples $\mu (\boldsymbol{x})$, and we report the optimal outcome as the representative entry for the table. For a fair comparison between enhanced EPR and standalone HJB loss, we maintain consistent network training configurations. Additionally, a detailed analysis of λ_1_ and μ(***x***) in Supplementary Section F.1 highlights enhanced EPR’s superior performance and robustness. Concerning the flow model, we leverage the same SDE-simulated dataset as utilized for enhanced EPR. The metrics presented in Table 1 yield the same consistent result, reinforcing the superiority of enhanced EPR over other methods across all problems.

**Table 1.** Comparisons of different methods. We report the mean and the standard deviation over five random seeds. The best results are highlighted in bold.

Problem	Method	rRMSE	rMAE
Toy, *D* = 0.1	Enhanced EPR	**0.028 ± 0.010**	**0.026 ± 0.010**
	HJB loss alone	0.034 ± 0.016	0.029 ± 0.014
	Normalizing flow	0.124 ± 0.016	0.082 ± 0.011
Toy, *D* = 0.05	Enhanced EPR	**0.054 ± 0.020**	**0.048 ± 0.019**
	HJB loss alone	0.191 ± 0.218	0.160 ± 0.179
	Normalizing flow	0.239 ± 0.040	0.174 ± 0.034
Multi-stable	Enhanced EPR	**0.067 ± 0.047**	**0.066 ± 0.049**
	HJB loss alone	0.249 ± 0.015	0.228 ± 0.011
	Normalizing flow	0.202 ± 0.029	0.133 ± 0.017
Limit cycle	Enhanced EPR	**0.070 ± 0.016**	**0.063 ± 0.020**
	HJB loss alone	0.231 ± 0.048	0.140 ± 0.021
	Normalizing flow	0.384 ± 0.021	0.267 ± 0.013

**Figure 3.**
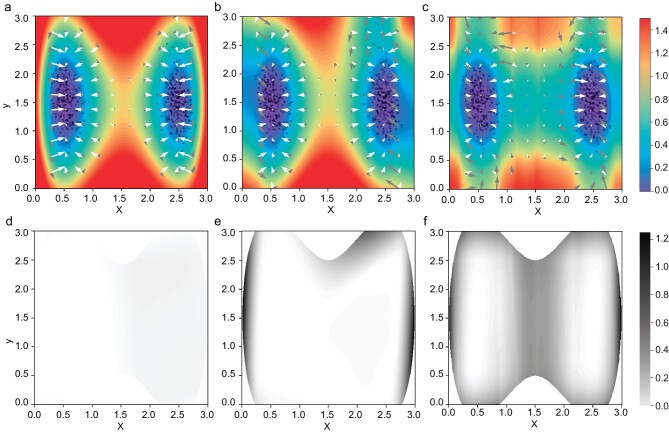
Comparisons between models learned by (a, d) enhanced EPR, (b, e) HJB loss alone and (c, f) normalizing flow. (a–c) Filled contour plots of the potential $V(\boldsymbol{x};\theta ^{*})$ for the toy model with *D* = 0.05. The force field $\boldsymbol{F}(\boldsymbol{x})$ is decomposed into the gradient part $-\nabla V(\boldsymbol{x};\theta ^{*})$ (white arrows) and the non-gradient part (gray arrows). The length of an arrow denotes the scale of the vector. The solid dots are samples from the simulated invariant distribution. The results in the high-energy region $\lbrace \boldsymbol{x} | V(\boldsymbol{x}) \ge 30D\rbrace$ are omitted since they are less relevant to the dynamics. (d–f) The absolute error of the learned potential constructed in different ways.

We further illustrate the learned potential landscapes obtained through different methods for the 2D toy model with *D* = 0.05 in Fig. 3a–c. Furthermore, we plot the absolute error between the learned potential and the reference solution in Fig. 3d–f. These plots demonstrate that errors within enhanced EPR are consistently small. In contrast, errors in HJB loss alone and NF can be significantly large, leading to unfavorable outcomes and limited smoothness. Based on this study and our numerical experiences, the advantages of enhanced EPR over both the approach using HJB loss alone and the approach using NF can be summarized as follows.

• *Accuracy.* As shown in Table 1, enhanced EPR achieves the best accuracy over the HJB loss alone and normalizing flow approaches in terms of the rRMSE and rMAE metrics. From Fig. 3, we also find that enhanced EPR presents an overall landscape more consistent with the simulated samples and the true/reference solution than the other two methods. The decomposition of the force also shows a better matching result. The learned potential by HJB loss alone and normalizing flow tends to be rough and non-smooth near the edge of samples (Fig. 3b and c). Compared with enhanced EPR and HJB loss alone, the normalizing flow captures the high-probability domain, but does not explicitly take advantage of the information of the dynamics, thus making its performance the worst.
• *Robustness.* Without the guidance of EPR loss, minimizing HJB loss alone does not lead to a good approximation of the true solution that can closely match the heuristically chosen distribution, as shown in Fig. 3b. The significant variance observed in the results for the toy model with *D* = 0.05 by solving HJB loss alone is attributed to an exceptionally poor outlier. However, enhanced EPR always gives reliable approximations with the introduced setup. This supports the robustness of enhanced EPR loss. We refer the reader to Supplementary Section F.1 for a detailed analysis of the chosen parameters λ_1_ and the distribution of the enhanced samples $\mu (\boldsymbol{x})$.
• *Efficiency.* Our numerical experiments show that enhanced EPR converges faster than the approach using HJB loss alone. For instance, in the toy model with *D* = 0.05, enhanced EPR achieves an rRMSE of 0.088 ± 0.083 and an rMAE of 0.075 ± 0.070 in 2000 epochs, while training with HJB loss alone cannot attain the same accuracy level even after 3000 epochs. As we have analyzed, this efficiency might be attributed to the strict convexity of EPR loss. Related theoretical support for this speculation can be found in [38].
• *Performance of single EPR.* We remark that single EPR which relies solely on EPR loss by setting λ = 0 in (15), can still give acceptable results with a relatively small *D* = 0.05. Its rRMSE and rMAE are 0.103 ± 0.075 and 0.087 ± 0.063, respectively, which are also small enough. In the limit-cycle problem, single EPR is not as good as in the toy model and the multi-stable problem, since there are much fewer samples in the domain where the non-gradient force is extremely large, as shown in Fig. 2b. However, single EPR loss can achieve competitive performance as long as the samples effectively cover the domain.

### Three-dimensional Lorenz system

We apply our landscape construction approach to the 3D Lorenz system [42] with isotropic temporal Gaussian white noise i.e. system (1) with $\boldsymbol{F}=(F_x,F_y,F_z)$ and


**Figure 4.**
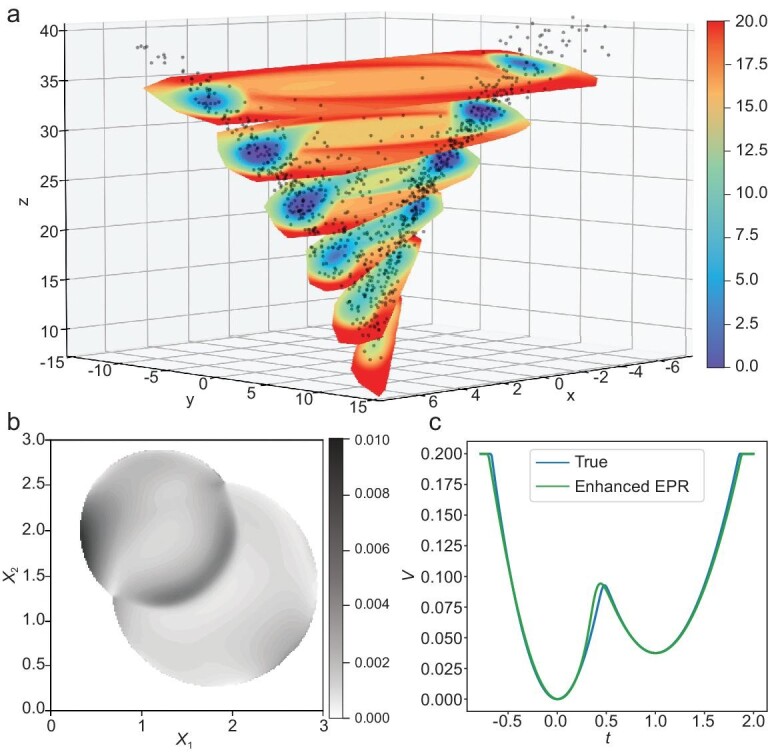
Landscapes constructed with enhanced EPR. (a) Slices of the learned 3D potential ${V}(\boldsymbol{x};\theta ^{*})$ in the Lorenz system. The filled circles are samples from the simulated invariant distribution. (b) The accumulated measure of the absolute error between $U_0(\boldsymbol{x})$ and the learned solution $V(\boldsymbol{x}; \theta ^{*})$ based on the conditional probability $p_0(\boldsymbol{x} |x_1, x_2)$. (c) The learned 12D potential $V(\boldsymbol{x}; \theta ^{*})$ along a line $\boldsymbol{x}(t)=t \boldsymbol{\mu }_1 + (1-t) \boldsymbol{\mu }_2$, where $\boldsymbol{\mu }_1,\boldsymbol{\mu }_2$ are the means of two components in a 12D Gaussian mixture.

(19) \begin{eqnarray*}
F_x(x, y, z) = \beta _1(y-x),
\end{eqnarray*}

(20) \begin{eqnarray*}
F_y(x, y, z) =x(\beta _2-z)-y,
\end{eqnarray*}

(21) \begin{eqnarray*}
F_z(x, y, z) =x y-\beta _3 z,
\end{eqnarray*}

where β_1_ = 10, β_2_ = 28 and $\beta _3=\frac{8}{3}$. We add noise with strength *D* = 1. This model was also considered in [23] with *D* = 20. We obtain the enhanced data $(\boldsymbol{x}^{\prime }_i)_{1\le i\le N^{\prime }}$ by adding Gaussian noise with standard deviation σ = 5 to the SDE-simulated data $(\boldsymbol{x}_i)_{1\le i\le N}$ where $N=N^{\prime }=10\, 000$. We directly train the 3D potential $V(\boldsymbol{x};\theta )$ by enhanced EPR (16) with λ_1_ = 10.0, λ_2_ = 1.0, using Adam with a learning rate of 0.001 and a batch size of 2048. A slice view of the landscape is presented in Fig. 4a and the learned 3D potential agrees well with the simulated samples.

### Twelve-dimensional multi-stable system

To further validate the efficacy of EPR-Net in high-dimensional scenarios, we construct a Gaussian mixture model (GMM) in $\mathbb {R}^{12}$ with two centers, of which the true solution can be denoted $U_0(\boldsymbol{x}) = -D \log p_0(\boldsymbol{x})$, where $p_0(\boldsymbol{x}) = w_1 p_1(\boldsymbol{x}) + w_2 p_2(\boldsymbol{x})$ and $p_i(\boldsymbol{x}) = Z_i^{-1}\exp (-(\boldsymbol{x}-\boldsymbol{\mu }_i)^\top \Sigma _i^{-1}(\boldsymbol{x}-\boldsymbol{\mu }_i)/2),\,\, i\,=\,1, 2$, with normalization factor *Z_i_*. The parameters are chosen as *w*_1_ = 0.6, *w*_2_ = 0.4, $\boldsymbol{\mu }_1 = (1.2, 2.0, 0.6, 1.5, 0.9, 1.5, 1.5, 0.9, 1.2, 1.2, 0.5$, 1.8)^⊤^, $\boldsymbol{\mu }_2 = (1.8, 1.4, 0.8, 0.9, 0.9, 1.5, 2.0, 1.0, 1.6, 1.0, 0.7, 1.4)^\top$, Σ_1_ = 0.04*I* and Σ_2_ = 0.02*I*.

For evaluation, we first sample 10 000 points from the known GMM distribution and calculate the relative error between the learned 12D potential $V(\boldsymbol{x}; \theta ^{*})$ and the true potential $U_0(\boldsymbol{x})$. The resulting rRMSE and rMAE are 0.054 and 0.051, respectively, indicating the high accuracy of the learned potential. In addition, we denote the first two dimensions of this 12D problem as (*x*_1_, *x*_2_) and draw samples from the corresponding conditional distribution $p_0(\boldsymbol{x} |x_1, x_2)$. As depicted in Fig. 4b, we compute the cumulative measure of the absolute error between $U_0(\boldsymbol{x})$ and the learned solution $V(\boldsymbol{x})$ based on the conditional distribution as $\int |U_0(\boldsymbol{x}) - V(\boldsymbol{x})| p_0(\boldsymbol{x} |x_1, x_2) {\rm d} \boldsymbol{x}$. Impressively, this integral consistently remains below 0.01, underscoring the fact that our approach yields negligible errors in the effective domain. Moreover, comparing the barrier heights (BHs) further validates the precision of our approach, with a relative error of less than 5% for both BHs. Because of the diagonal covariance matrix, the saddle point precisely lies on the line passing through $\boldsymbol{\mu }_1$ and $\boldsymbol{\mu }_2$, allowing us to directly compare the BHs. As shown in Fig 4c, the true and computed slice lines coincide well. For additional details, see Supplementary Section F.5.

## DIMENSIONALITY REDUCTION

When applying the approach above to high-dimensional problems, dimensionality reduction is necessary in order to visualize the results and gain physical insights. For simplicity, we consider the linear case and, with a slight abuse of notation, let $\boldsymbol{x} = (\boldsymbol{y}, \boldsymbol{z})^\top$, where $\boldsymbol{z} = (x_i, x_j) \in \mathbb {R}^2$ contains the coordinates of two variables of interest, and $\boldsymbol{y} \in \mathbb {R}^{d-2}$ corresponds to the remaining *d* − 2 variables. The domain Ω (either $\mathbb {R}^d$ or a *d*-dimensional hyperrectangle) has the decomposition $\Omega =\Sigma \times \widetilde{\Omega }$, where $\Sigma \subseteq \mathbb {R}^{d-2}$ and $\widetilde{\Omega }\subseteq \mathbb {R}^2$ are the domains of $\boldsymbol{y}$ and $\boldsymbol{z}$, respectively. Automatic selection of linear reduced variables and extensions to nonlinear reduced variables with general domains are also possible [43,44]. In the current setting, the reduced potential is


(22) \begin{eqnarray*}
\widetilde{U}(\boldsymbol{z}) = -D\ln \widetilde{p}_{\mathrm{ss}}(\boldsymbol{z}) = -D\ln \int _{\Sigma } p_{\mathrm{ss}}(\boldsymbol{y}, \boldsymbol{z})\, {\rm d} \boldsymbol{y},
\end{eqnarray*}

and one can show that $\widetilde{U}$ minimizes the loss function


(23) \begin{eqnarray*}
{\rm L}_{ \hbox{P-EPR}}(\widetilde{V})\!=\!\int _{\Omega }|\boldsymbol{F}_{\boldsymbol{z}}(\boldsymbol{y}, \boldsymbol{z})\!+\!\nabla _{\boldsymbol{z}} \widetilde{V}(\boldsymbol{z};\theta )|^2 \, {\rm d} \pi (\boldsymbol{y}, \boldsymbol{z}),
\end{eqnarray*}

where $\boldsymbol{F}_{\boldsymbol{z}}(\boldsymbol{y}, \boldsymbol{z})\in \mathbb {R}^{2}$ is the $\boldsymbol{z}$ component of the force field $\boldsymbol{F}=(\boldsymbol{F}_{\boldsymbol{y}}, \boldsymbol{F}_{\boldsymbol{z}})^\top$.

Moreover, one can derive an enhanced loss as in (15) that could be used for systems with small *D*. To this end, we note that $\widetilde{U}$ satisfies the projected HJB equation


(24) \begin{eqnarray*}
\mathcal {N}_{\textrm {P-HJB}}(\widetilde{U})&:=& -\widetilde{\boldsymbol{F}} \cdot \nabla _{\boldsymbol{z}} \widetilde{U} + D \Delta _{\boldsymbol{z}} \widetilde{U} -|\nabla _{\boldsymbol{z}} \widetilde{U}|^2\\
&& +\, D \nabla _{\boldsymbol{z}} \cdot \widetilde{\boldsymbol{F}} =0,
\end{eqnarray*}

with asymptotic BC $\widetilde{U}\rightarrow \infty$ as $|\boldsymbol{z}|\rightarrow \infty$, or reflecting BC $(\widetilde{\boldsymbol{F}}+\nabla _{\boldsymbol{z}} \widetilde{U}) \cdot \widetilde{\boldsymbol{n}}=0$ on $\partial \widetilde{\Omega }$, where $\widetilde{\boldsymbol{F}}(\boldsymbol{z}):=\int _{\Sigma } \boldsymbol{F}_{\boldsymbol{z}}(\boldsymbol{y}, \boldsymbol{z}) {\rm d} \pi (\boldsymbol{y}|\boldsymbol{z})$ is the projected force defined using the conditional distribution ${\rm d} \pi (\boldsymbol{y}|\boldsymbol{z}) = p_{\mathrm{ss}}(\boldsymbol{y},\boldsymbol{z})/\widetilde{p}_{\mathrm{ss}}(\boldsymbol{z})\, {\rm d} \boldsymbol{y}$ and $\widetilde{\boldsymbol{n}}$ denotes the unit outer normal on $\partial \widetilde{\Omega }$. Based on (24), we can formulate the projected HJB loss


(25) \begin{eqnarray*}
{\rm L}_{\hbox{P-HJB}}(\widetilde{V})=\int _{\widetilde{\Omega }} |\mathcal {N}_{\textrm {P-HJB}}(\widetilde{V})|^2 \, {\rm d} \mu (\boldsymbol{z}),
\end{eqnarray*}

where μ is any suitable distribution over $\widetilde{\Omega }$, and $\widetilde{\boldsymbol{F}}$ in (24) is learned beforehand by training a DNN with the loss


(26) \begin{eqnarray*}
{\rm L}_{\hbox{P-For}}(\widetilde{\boldsymbol{G}})=\int _{\Omega }|\boldsymbol{F}_{\boldsymbol{z}}(\boldsymbol{y}, \boldsymbol{z})- \widetilde{\boldsymbol{G}}(\boldsymbol{z}; \theta )|^2 \, {\rm d} \pi (\boldsymbol{y}, \boldsymbol{z}).
\end{eqnarray*}

The overall enhanced loss (16) used in numerical computations comprises two terms, which are empirical estimates of (23) and (25) based on two different sets of sample data. We refer the reader to the online supplementary material for derivation details (Section C), experiments of Ferrell’s cell cycle model [45] (Section G.1) and for details and additional results of the 8D [4] and 52D [27] models (Sections G.2 and G.3).

### Eight-dimensional cell cycle: reduced potential

**Figure 5.**
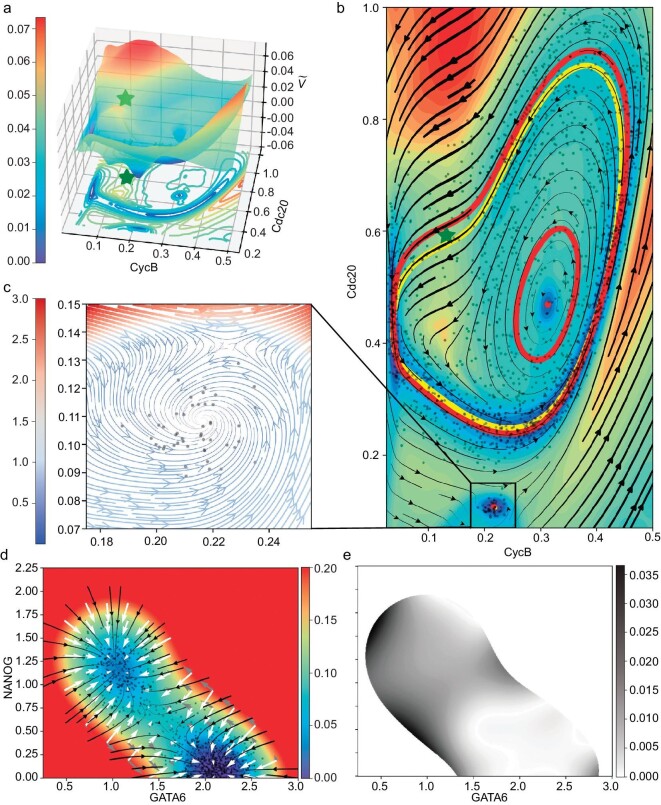
Dimensionality reduction of high-dimensional systems. (a–c) Eight-dimensional cell cycle model. (a) Reduced potential landscape $\widetilde{V}$ with projected contour lines. The star at (0.13, 0.59) denotes the saddle point of $\widetilde{V}$. (b) Projected sample points, streamlines of the projected force field $\widetilde{\boldsymbol{G}}(\boldsymbol{x};\theta ^{*})$ and the filled contour plot of $\widetilde{V}(\boldsymbol{x};\theta ^{*})$. Two red circles and two red dots (close to (0.22,0.11) and (0.31,0.47), respectively) show the stable limit sets of the projected force field. The yellow circle is the projection of the original high-dimensional limit cycle. (c) An enlarged view of the square domain in (b), showing the detailed spiral structure of the streamlines of $\widetilde{\boldsymbol{G}}(\boldsymbol{x};\theta ^{*})$ around the stable point. (d and e) Fifty-two-dimensional multi-stable system. (d) Projected force $\widetilde{\boldsymbol{G}}(\boldsymbol{z};\theta ^{*})$ and potential $\widetilde{V_1}(\boldsymbol{z};\theta ^{*})$ of the 52D double-well model learned by enhanced EPR. (e) The absolute error of the reduced potential constructed in different ways, i.e. $|\widetilde{V_1}(\boldsymbol{z};\theta ^{*}) - \widetilde{V_2}(\boldsymbol{z};\theta ^{*})|$.

Subsequently we apply our dimensionality reduction approach to construct the landscape for an 8D cell cycle model [4] which contains both a limit cycle and a stable equilibrium point. In this case, we consider CycB (a cyclin B protein) and Cdc20 (an exit protein) as the reduced variables following [4]. As shown in Fig. 5a and b we can find that both the profile of the reduced potential and the force strength agree well with the density of projected samples. Moreover we gain some important insights from Fig. 5b on the projection of the high-dimensional dynamics to two dimensions. One particular feature is that the limit cycle induced by the projected force $\widetilde{\boldsymbol{G}}(\boldsymbol{x};\theta ^{*})$ (outer red circle) slightly differs from the limit cycle directly projected from high dimensions (yellow circle), and the difference is either minor or moderate depending on whether the sample density near the limit circle is high or low. This is natural in the reduction when *D* > 0, since the distribution $\pi (\boldsymbol{y}|\boldsymbol{z})$ involved in computing $\widetilde{\boldsymbol{G}}(\boldsymbol{x};\theta ^{*})$ is not a Dirac-δ distribution, but a diffusive distribution with varying widths along the limit cycle, and the difference will disappear as *D* → 0. Another feature is that we unexpectedly get an additional stable limit cycle (inner red circle) and a stable point (red dot in the center) emerging inside the outer limit cycle. Though virtual in high dimensions and biologically irrelevant, the existence of such limit sets is reminiscent of the Poincaré–Bendixson theorem in planar dynamics theory (Chapter 10.6 of [46]), which depicts a common phenomenon when performing dimensionality reduction with limit cycles to the 2D plane. Note that this theorem does not guarantee the stability of such fixed points; they could be stable, unstable or even saddle points; see another case in Supplementary Section G.1. The emergence of these two limit sets is specific in this model due to the relatively flat landscape of the potential in the centering region. In addition, close to the saddle point of $\widetilde{V}$ (green star), there is a barrier along the limit-cycle direction, while there is a local well along the Cdc20 direction, which characterizes the region that biological cycle paths mainly go through. Last but not least, an enlarged view of the local attractive domain outside the limit cycle shows its intricate spiral structure (Fig. 5c), which has not been revealed in previous work based on mean-field approximation [4].

### Fifty-two-dimensional multi-stable system: high-dimensional and reduced potentials

We compare two approaches to construct the reduced potential. One is to learn the reduced force $\widetilde{\boldsymbol{G}}(\boldsymbol{z}; \theta ^{*})$ by (26) first and then use it to construct the landscape $\widetilde{V}(\boldsymbol{z}; \theta ^{*})$ by enhanced EPR in $\boldsymbol{z}$ space. The other is to build a high-dimensional potential $V(\boldsymbol{x}; \theta ^{*})$ by enhanced EPR first and then reduce it (see Supplementary Section C.1. For this comparison, we apply our approach to a gene stem cell regulatory network described by an ordinary differential equation (ODE) in 52 dimensions [27] and take GATA6 (a major differentiation marker gene) and NANOG (a major stem cell marker gene) as the reduced variable $\boldsymbol{z}$ as suggested in [27]. We define $\mathcal {A}_i$ to be the set of indices for activating *x_i_* and $\mathcal {R}_i$ to be the set of indices for repressing *x_i_*; the corresponding relationships are defined as the 52D node network shown in [27]. For *i* = 1, …, 52,


(27) \begin{eqnarray*}
F_i(\boldsymbol{x}) = -k x_i+\sum _{j\in \mathcal {A}_j}^{} \frac{a x_j^n}{S^n+x_j^n}+\sum _{j\in \mathcal {R}_j}^{} \frac{b S^n}{S^n+x_j^n},
\end{eqnarray*}

where *a* = 0.37, *b* = 0.5, *k* = 1, *S* = 0.5 and *n* = 3. We choose the noise strength *D* = 0.02 and focus on the domain Ω = [0, 3]^52^.

As shown in Fig. 5d, the projected force $\widetilde{\boldsymbol{G}}(\boldsymbol{z};\theta ^{*})$ demonstrates the reduced dynamics and the constructed potential $\widetilde{V_1}(\boldsymbol{z};\theta ^{*})$ agrees well with the SDE-simulated samples. While $\widetilde{V_1}(\boldsymbol{z};\theta ^{*})$ is learned by first obtaining the projected force, $\widetilde{V_2}(\boldsymbol{z};\theta ^{*})$ is reduced from a high-dimensional ${V}(\boldsymbol{x};\theta ^{*})$ precomputed by enhanced EPR. The gray plot of the absolute error of $|\widetilde{V_1}(\boldsymbol{z};\theta ^{*}) - \widetilde{V_2}(\boldsymbol{z};\theta ^{*})|$ is shown in Fig. 5e, which supports the consistency of two potentials learned by different approaches. When taking $\widetilde{V_1}(\boldsymbol{z};\theta ^{*})$ as the reference solution, we get the rRMSE 0.113 and rMAE 0.097. The minor relative errors show that these two approaches to constructing the reduced potential are quantitatively consistent, although it is difficult to know which is more accurate. It also indirectly supports the reliability of the learned high-dimensional potential $V(\boldsymbol{x}; \theta ^{*})$. The obtained landscape shows a more smooth and more delicate profile compared to the mean-field approach [27].

## DISCUSSION AND CONCLUSION

The EPR-Net formulation can be extended to the case of state-dependent diffusion coefficients without difficulty. Consider the Itô SDE


(28) \begin{eqnarray*}
\frac{{\rm d} \boldsymbol{x}(t)}{{\rm d} t}=\boldsymbol{F}(\boldsymbol{x}) + \sqrt{2D}\sigma (\boldsymbol{x})\ \dot{\boldsymbol{w},}\qquad \boldsymbol{x}(0)=\boldsymbol{x}_0,
\end{eqnarray*}

with diffusion matrix $\sigma (\boldsymbol{x})\in \mathbb {R}^{d\times m}$ and $\dot{\boldsymbol{w}}$ an *m*-dimensional temporal Gaussian white noise. We assume that *m* ≥ *d* and that the matrix $a(\boldsymbol{x}):=(\sigma \sigma ^\top )(\boldsymbol{x})$ satisfies $\boldsymbol{u}^\top a(\boldsymbol{x})\boldsymbol{u} \ge c_0 |\boldsymbol{u}|^2$ for all $\boldsymbol {x}, \boldsymbol{u}\in \mathbb {R}^d$, where *c*_0_ > 0 is a positive constant. Using a similar derivation as before, we can again show that the high-dimensional landscape function *U* of (28) minimizes the EPR loss


(29) \begin{eqnarray*}
{\rm L}_{\hbox{V-EPR}}(V) = \int _{\Omega }|\boldsymbol{F}^v(\boldsymbol{x})+ a(\boldsymbol{x}) \nabla V(\boldsymbol{x})|_{a^{-1}}^2 \, {\rm d} \pi {(\boldsymbol{x})},
\end{eqnarray*}

where $\boldsymbol{F}^v(\boldsymbol{x}) = \boldsymbol{F}(\boldsymbol{x}) - D \nabla \cdot a(\boldsymbol{x})$ and $|\boldsymbol{u}|_{a^{-1}}^2:=\boldsymbol{u}^\top a^{-1}(\boldsymbol{x})\boldsymbol{u}$ for $\boldsymbol{u}\in \mathbb {R}^d$. We provide derivation details of (29) in Section D within the online supplementary material, and we leave the numerical study of (28) and (29) to future work.

To summarize, we have demonstrated the formulation, applicability and superiority of EPR to the benchmark and real biological examples over some existing approaches. Below we make some final remarks. First, concerning the use of the steady-state distribution $\pi (\boldsymbol{x})$ in (3) and its approximation by a long time series of SDE (1) in EPR-Net, we emphasize that it is the sampling approximation of π that naturally captures the important parts of the potential function, and the landscape beyond the sampled regions is not that essential in practice. Second, as is exemplified in Fig. 2 and Table 1, we found that a direct application of density estimation methods (DEMs), e.g. normalizing flows [39], to the sampled time series data does not give potential landscapes with satisfactory accuracy. We speculate that such deficiency of the DEM is due to its over-generality and the fact that it does not take advantage of the force field information explicitly compared to (3). Finally, we remark that in our dimensionality reduction approach, we choose the reduced variables according to available biophysics prior knowledge. Our approach also exhibits the capability to be extended to incorporate a state-dependent diffusion coefficient. Automatic selection of general nonlinear reduced variables can also be studied by incorporating the idea of the identification of collected variables in molecular simulations [47,48].

Overall, we have presented the EPR-Net, a simple yet effective DNN approach, for constructing the non-equilibrium potential landscape of NESS systems. This approach is both elegant and robust due to its variational structure and its flexibility to be combined with other types of loss functions. Further extensions of dimensionality reduction to nonlinear reduced variables and numerical investigations in the case of state-dependent diffusion coefficients will be explored in future research. Moreover, we are actively considering the application of our method to single-cell RNA sequencing data, which involves systems of really high dimensionality and typically lacks analytical ODE formulations. We will explore this point in our forthcoming studies.

## METHODS

Detailed methods are given in the online supplementary material. The code is accessible at https://github.com/yzhao98/EPR-Net.

## Supplementary Material

nwae052_Supplemental_Files
