# Supplementary material for: EPR-Net: constructing a non-equilibrium potential landscape via a variational force projection formulation
Source: Natl Sci Rev. 2024 Feb 20;11(7):nwae052. doi: 10.1093/nsr/nwae052 (PMC11173252; doi:10.1093/nsr/nwae052)
Supplement: nwae052_Supplemental_Files [file nwae052_supplemental_files.zip › supplementary information.pdf]

Supplementary Information for EPR-Net: Constructing non-equilibrium potential landscape via a variational force projection formulation

Yue Zhao[1], Wei Zhang[2,3*], Tiejun Li[1,4,5*]

[1*]Center for Data Science, Peking University, No. 5 Yiheyuan Road, Beijing, 100871, China.
[2]Zuse Institute Berlin, Takustrasse 7, 14195 Berlin, Germany.
[3]Department of Mathematics and Computer Science, Freie Universität Berlin, Arnimallee 6, 14195 Berlin, Germany.
[4]LMAM and School of Mathematical Sciences, Peking University, No. 5 Yiheyuan Road, Beijing, 100871, China.
[5]Center for Machine Learning Research, Peking University, No. 5 Yiheyuan Road, Beijing, 100871, China.

*Corresponding author(s). E-mail(s): wei.zhang@fu-berlin.de; tieli@pku.edu.cn;
Contributing authors: zhaoyue@stu.pku.edu.cn;

Contents

Part 1: Theory 3

A Derivation of the EPR loss 3

B Stability of the EPR minimizer 4

C Dimensionality reduction 5
- C.1 Gradient projection loss . . . 5
- C.2 Projected EPR loss . . . 6
- C.3 Force projection loss . . . 7
- C.4 HJB equation for the reduced potential . . . 8

D State-dependent diffusion coefficients 9

Part 2: Computation 11

E Motivation for enhanced EPR 11

F Landscape with original coordinates 12
- F.1 Training details and additional results for benchmark problems . . . 12
- F.2 2D toy model . . . 14
- F.3 2D limit cycle model . . . 15
- F.4 2D multi-stable model . . . 16
- F.5 12D GMM model: high-dimensional potential . . . 16

G Landscape with reduced coordinates 16
- G.1 Ferrell's three-ODE model: reduced potential . . . 16
- G.2 8D complex system: reduced potential . . . 18
- G.3 52D multi-stable system: high-dimensional and reduced potentials . . . 18

In this supplementary information (SI), we will present further theoretical derivations and computational details of the contents in the main text (MT). This SI consists of two parts: *theory* and *computation*.

PART 1: THEORY

We will first provide details of theoretical derivations omitted in the MT.

A Derivation of the EPR loss

In this section, we show that, up to an additive constant, the potential function $U(\boldsymbol{x}) := -D \ln p_{\mathrm{ss}}(\boldsymbol{x})$ is the unique minimizer of the EPR loss in equation (1) in MT.

First, we show that the orthogonality relation

$$\int_\Omega (\boldsymbol{F} + \nabla U) \cdot \nabla W \,\mathrm{d}\pi = 0 \tag{1}$$

holds for any suitable function $W(\boldsymbol{x}) : \mathbb{R}^d \to \mathbb{R}$ under both choices of the boundary conditions considered in this work, where $\mathrm{d}\pi(\boldsymbol{x}) := p_{\mathrm{ss}}(\boldsymbol{x})\mathrm{d}\boldsymbol{x}$. To see this, we note that

$$\begin{aligned}
&\int_\Omega (\boldsymbol{F} + \nabla U) \cdot \nabla W \,\mathrm{d}\pi \\
=&\int_\Omega (p_{\mathrm{ss}}\boldsymbol{F} - D\nabla p_{\mathrm{ss}}) \cdot \nabla W \,\mathrm{d}\boldsymbol{x} \\
=&\int_{\partial\Omega} W(p_{\mathrm{ss}}\boldsymbol{F} - D\nabla p_{\mathrm{ss}}) \cdot \boldsymbol{n} \,\mathrm{d}\boldsymbol{x} \\
&- \int_\Omega W\nabla \cdot (p_{\mathrm{ss}}\boldsymbol{F} - D\nabla p_{\mathrm{ss}}) \,\mathrm{d}\boldsymbol{x} \\
=&0
\end{aligned}$$

where we have used integration by parts, the relation $p_{\mathrm{ss}}(\boldsymbol{x}) = \exp(-U(\boldsymbol{x})/D)$, the corresponding BC, and the steady state Fokker-Planck equation (FPE) satisfied by p_{ss}.

Now consider the EPR loss, we have

$$\begin{aligned}
\mathrm{L}_{\mathrm{EPR}}(V) &= \int_\Omega |\boldsymbol{F} + \nabla V|^2 \,\mathrm{d}\pi \\
&= \int_\Omega |\boldsymbol{F} + \nabla U|^2 + |\nabla V - \nabla U|^2 \,\mathrm{d}\pi,
\end{aligned}$$

where we have used the orthogonality relation equation (10) to arrive at the last equality, from which we conclude that $U(\boldsymbol{x})$ is the unique minimizer of the EPR loss up to an additive constant.

B Stability of the EPR minimizer

In this section, we formally show that small perturbations of the invariant distribution π will not introduce a disastrous change to the minimizer of the corresponding EPR loss. We only consider the case where Ω is a d-dimensional hyperrectangle. The argument for $\Omega = \mathbb{R}^d$ is similar.

Suppose $\mathrm{d}\pi(\boldsymbol{x}) = p(\boldsymbol{x})\mathrm{d}\boldsymbol{x}$, $\mathrm{d}\mu(\boldsymbol{x}) = q(\boldsymbol{x})\mathrm{d}\boldsymbol{x}$, and the functions $U(\boldsymbol{x})$ and $\bar{U}(\boldsymbol{x})$ are the unique minimizers (up to a constant) of the following two EPR losses

$$
\begin{aligned}
U &= \arg\min_V \int_\Omega |\boldsymbol{F} + \nabla V|^2 \,\mathrm{d}\pi, \\
\bar{U} &= \arg\min_V \int_\Omega |\boldsymbol{F} + \nabla V|^2 \,\mathrm{d}\mu,
\end{aligned}
$$

respectively. It is not difficult to find that the Euler-Lagrange equations of $U, \bar{U}$ are given by the following partial differential equation (PDE) with suitable BCs:

$$
\begin{aligned}
\nabla \cdot ((\boldsymbol{F} + \nabla U)p) = 0 \text{ in } \Omega, \ (\boldsymbol{F} + \nabla U) \cdot \boldsymbol{n} = 0 \text{ on } \partial\Omega, \\
\nabla \cdot ((\boldsymbol{F} + \nabla \bar{U})q) = 0 \text{ in } \Omega, \ (\boldsymbol{F} + \nabla \bar{U}) \cdot \boldsymbol{n} = 0 \text{ on } \partial\Omega.
\end{aligned}
$$

The PDEs above defined inside the domain Ω can be converted to

$$
\begin{aligned}
\Delta U p + \nabla U \cdot \nabla p = -\nabla \cdot (p\boldsymbol{F}), \\
\Delta \bar{U} q + \nabla \bar{U} \cdot \nabla q = -\nabla \cdot (q\boldsymbol{F}).
\end{aligned}
$$

Define $U_0(\boldsymbol{x}) = -D \ln p(\boldsymbol{x})$ and $\bar{U}_0(\boldsymbol{x}) = -D \ln q(\boldsymbol{x})$. We then obtain

$$
-\nabla U \cdot \nabla U_0 + D\Delta U = \boldsymbol{F} \cdot \nabla U_0 - D\nabla \cdot \boldsymbol{F}, \tag{2}
$$

$$
-\nabla \bar{U} \cdot \nabla \bar{U}_0 + D\Delta \bar{U} = \boldsymbol{F} \cdot \nabla \bar{U}_0 - D\nabla \cdot \boldsymbol{F}. \tag{3}
$$

Assuming that $\delta U_0 := U_0 - \bar{U}_0 = O(\varepsilon)$, where $0 < \epsilon \ll 1$ denotes a small constant, we have the PDE for $U - \bar{U}$ by subtracting equation (3) from equation (2):

$$
-\nabla(U - \bar{U}) \cdot \nabla U_0 + D\Delta(U - \bar{U}) = \boldsymbol{F} \cdot \nabla(\delta U_0) + \nabla \bar{U} \cdot \nabla(\delta U_0)
$$

with BC $\nabla(U - \bar{U}) \cdot \boldsymbol{n} = 0$. Since $U_0, \bar{U}, \boldsymbol{F} \sim O(1)$, we can obtain that

$$
U(\boldsymbol{x}) - \bar{U}(\boldsymbol{x}) = O(\varepsilon)
$$

by the regularity theory of elliptic PDE [1, Section 6.3] when $D \sim O(1)$, or by the matched asymptotic expansion when $D \ll 1$ [2, Chapter 2]. In fact, the closeness between $U(\boldsymbol{x})$ and $\bar{U}(\boldsymbol{x})$ can be ensured as long as U_0 and $\bar{U}_0$ are close enough in the region where $p(\boldsymbol{x})$ and $q(\boldsymbol{x})$ are bounded away from zero by the method of characteristics analysis [1, Section 2.1] and matched asymptotics.

The above derivations assume that the PDFs p,q are smooth enough. The stability analysis for general distributions π and μ is also possible by utilizing the functional analysis tools. However, the derivations will be abstract and quite involved, and we will report it elsewhere.

C Dimensionality reduction

In this section, we study dimensionality reduction for high-dimensional problems in order to learn the projected potential. A straightforward approach is to first learn the high-dimensional potential U and then find its low-dimensional representation, i.e., the reduced potential or the free energy function, using dimensionality reduction techniques. An alternative approach is to directly learn the low-dimensional reduced potential.

Denote by $\boldsymbol{x} = (\boldsymbol{y}, \boldsymbol{z})^\top \in \Omega$. As in MT Section 1.4, we assume the domain

$$\Omega = \Sigma \times \widetilde{\Omega},$$

where $\Sigma \subseteq \mathbb{R}^{d-2}$ and $\widetilde{\Omega} \subseteq \mathbb{R}^2$ are the domain of $\boldsymbol{y}$ and $\boldsymbol{z}$, respectively. The reduced potential $\widetilde{U}(\boldsymbol{z})$ is defined as

$$\widetilde{U}(\boldsymbol{z}) = -D \ln \widetilde{p}_{\mathrm{ss}}(\boldsymbol{z}) = -D \ln \int_\Sigma p_{\mathrm{ss}}(\boldsymbol{y}, \boldsymbol{z})\, \mathrm{d}\boldsymbol{y}. \tag{4}$$

One natural approach for constructing $\widetilde{U}(\boldsymbol{z})$ is directly integrating $p_{\mathrm{ss}}(\boldsymbol{y}, \boldsymbol{z})$ based on the learned $U(\boldsymbol{y}, \boldsymbol{z})$ with the EPR loss, i.e.,

$$\widetilde{U}(\boldsymbol{z}) = -D \ln \int_\Sigma \exp(-U(\boldsymbol{y}, \boldsymbol{z})/D)\, \mathrm{d}\boldsymbol{y}. \tag{5}$$

However, performing this integration is not a straightforward numerical task (see, e.g., [3, Chapter 7]).

C.1 Gradient projection loss

In this subsection, we study a simple approach to approximate $\widetilde{U}(\boldsymbol{z})$ based on sample points, which approximately obey the invariant distribution $\pi(\boldsymbol{x})$, and the learned high dimensional potential function $U(\boldsymbol{x})$ by EPR loss. This approach is taken in the consistency checking of the reduced potentials by different methods in SI Section G.3. The idea is to utilize the gradient projection (GP) loss on the $\boldsymbol{z}$ components of ∇U:

$$\mathrm{L}_{\mathrm{GP}}(\widetilde{V}) = \int_\Omega \left|\nabla_{\boldsymbol{z}} U(\boldsymbol{y}, \boldsymbol{z}) - \nabla_{\boldsymbol{z}} \widetilde{V}(\boldsymbol{z})\right|^2 \mathrm{d}\pi(\boldsymbol{y}, \boldsymbol{z}). \tag{6}$$

To justify equation (6), we note that

$$\mathrm{L}_{\mathrm{GP}}(\widetilde{V}) = \int_\Omega \left|\nabla_{\boldsymbol{z}} U - \nabla_{\boldsymbol{z}} \widetilde{V}\right|^2 \mathrm{d}\pi(\boldsymbol{x})$$

$$
\begin{aligned}
&= \int_{\Omega} \left|\nabla_{\boldsymbol{z}} U - \nabla_{\boldsymbol{z}} \widetilde{U} + \nabla_{\boldsymbol{z}} \widetilde{U} - \nabla_{\boldsymbol{z}} \widetilde{V}\right|^2 \mathrm{d}\pi(\boldsymbol{x}) \\
&= \int_{\Omega} \left(\left|\nabla_{\boldsymbol{z}} U - \nabla_{\boldsymbol{z}} \widetilde{U}\right|^2 + \left|\nabla_{\boldsymbol{z}} \widetilde{U} - \nabla_{\boldsymbol{z}} \widetilde{V}\right|^2\right) \mathrm{d}\pi(\boldsymbol{x}) \\
&\quad + 2 \int_{\Omega} (\nabla_{\boldsymbol{z}} U - \nabla_{\boldsymbol{z}} \widetilde{U}) \cdot \nabla_{\boldsymbol{z}} (\widetilde{U} - \widetilde{V}) \, \mathrm{d}\pi(\boldsymbol{x}) \\
&=: P_1 + P_2,
\end{aligned}
$$

where P_1 and P_2 denote the terms in the third and the fourth line above, respectively. The term $P_2 = 0$ since

$$
\begin{aligned}
&\int_{\Omega} \nabla_{\boldsymbol{z}} U \cdot \nabla_{\boldsymbol{z}} (\widetilde{U} - \widetilde{V}) \, \mathrm{d}\pi(\boldsymbol{x}) \\
&= \int_{\widetilde{\Omega}} \left(\int_{\Sigma} \nabla_{\boldsymbol{z}} U \mathrm{e}^{-\frac{U}{D}} \mathrm{d}\boldsymbol{y} \right) \cdot \nabla_{\boldsymbol{z}} (\widetilde{U} - \widetilde{V}) \, \mathrm{d}\boldsymbol{z} \\
&= - D \int_{\widetilde{\Omega}} \nabla_{\boldsymbol{z}} \left(\int_{\Sigma} \mathrm{e}^{-\frac{U}{D}} \, \mathrm{d}\boldsymbol{y} \right) \cdot \nabla_{\boldsymbol{z}} (\widetilde{U} - \widetilde{V}) \, \mathrm{d}\boldsymbol{z} \\
&= - D \int_{\widetilde{\Omega}} \nabla_{\boldsymbol{z}} \widetilde{p}_{\mathrm{ss}} \cdot \nabla_{\boldsymbol{z}} (\widetilde{U} - \widetilde{V}) \, \mathrm{d}\boldsymbol{z} \\
&= \int_{\widetilde{\Omega}} \nabla_{\boldsymbol{z}} \widetilde{U} \cdot \nabla_{\boldsymbol{z}} (\widetilde{U} - \widetilde{V}) \, \widetilde{p}_{\mathrm{ss}} \, \mathrm{d}\boldsymbol{z}
\end{aligned}
$$

and

$$
\int_{\Omega} \nabla_{\boldsymbol{z}} \widetilde{U} \cdot \nabla_{\boldsymbol{z}} (\widetilde{U} - \widetilde{V}) \, \mathrm{d}\pi(\boldsymbol{x}) = \int_{\widetilde{\Omega}} \nabla_{\boldsymbol{z}} \widetilde{U} \cdot \nabla_{\boldsymbol{z}} (\widetilde{U} - \widetilde{V}) \, \widetilde{p}_{\mathrm{ss}} \, \mathrm{d}\boldsymbol{z},
$$

which cancel with each other in P_2.

Therefore, the minimization of GP loss is equivalent to minimizing

$$
\int_{\widetilde{\Omega}} \left|\nabla_{\boldsymbol{z}} \widetilde{U} - \nabla_{\boldsymbol{z}} \widetilde{V}\right|^2 \widetilde{p}_{\mathrm{ss}} \, \mathrm{d}\boldsymbol{z},
$$

which clearly implies that $\widetilde{U}(\boldsymbol{z})$ is the unique minimizer (up to a constant) of the proposed GP loss.

C.2 Projected EPR loss

In this subsection, we study the projected EPR (P-EPR) loss, which has the form

$$
\mathrm{L}_{\text{P-EPR}}(\widetilde{V}) = \int_{\Omega} \left|\boldsymbol{F}_{\boldsymbol{z}}(\boldsymbol{y}, \boldsymbol{z}) + \nabla_{\boldsymbol{z}} \widetilde{V}(\boldsymbol{z})\right|^2 \mathrm{d}\pi(\boldsymbol{y}, \boldsymbol{z}), \tag{7}
$$

where $\boldsymbol{F}_{\boldsymbol{z}}(\boldsymbol{y}, \boldsymbol{z}) \in \mathbb{R}^2$ is the $\boldsymbol{z}$-component of the force field $\boldsymbol{F} = (\boldsymbol{F}_{\boldsymbol{y}}, \boldsymbol{F}_{\boldsymbol{z}})^\top$.

Define

$$\widetilde{\mathrm{L}}_{\text{P-EPR}}(\widetilde{V}) = \int_\Omega \left|\boldsymbol{F}(\boldsymbol{y},\boldsymbol{z}) + \nabla \widetilde{V}(\boldsymbol{z})\right|^2 \mathrm{d}\pi(\boldsymbol{y},\boldsymbol{z}), \tag{8}$$

where ∇ is the full gradient with respect to $\boldsymbol{x}$. To justify equation (7), we first note the following equivalence

$$\min \mathrm{L}_{\text{P-EPR}}(\widetilde{V}) \quad \Longleftrightarrow \quad \min \widetilde{\mathrm{L}}_{\text{P-EPR}}(\widetilde{V}), \tag{9}$$

since $\nabla_{\boldsymbol{y}} \widetilde{V}(\boldsymbol{z}) = 0$ and the $\boldsymbol{y}$-components of $\boldsymbol{F} + \nabla\widetilde{V}$ only introduce an irrelevant constant in equation (8). Furthermore, we have

$$\begin{aligned}
\widetilde{\mathrm{L}}_{\text{P-EPR}}(\widetilde{V}) &= \int_\Omega \left|\boldsymbol{F} + \nabla\widetilde{V}\right|^2 \mathrm{d}\pi(\boldsymbol{x}) \\
&= \int_\Omega \left|\boldsymbol{F} + \nabla U + \nabla\widetilde{V} - \nabla U\right|^2 \mathrm{d}\pi(\boldsymbol{x}) \\
&= \int_\Omega \left|\boldsymbol{F} + \nabla U\right|^2 + \left|\nabla\widetilde{V} - \nabla U\right|^2 \mathrm{d}\pi(\boldsymbol{x}),
\end{aligned}$$

where the last equality is due to the orthogonality relation equation (10).

$$\int_\Omega \big(\boldsymbol{F}(\boldsymbol{x}) + \nabla U(\boldsymbol{x})\big) \cdot \nabla W(\boldsymbol{x})\, \mathrm{d}\pi(\boldsymbol{x}) = 0, \tag{10}$$

Using a similar argument for deriving equation (9), the equivalence equation (9) itself, as well as the GP loss in equation (6), we get

$$\min \mathrm{L}_{\text{P-EPR}}(\widetilde{V}) \quad \Longleftrightarrow \quad \min \mathrm{L}_{\text{GP}}(\widetilde{V}). \tag{11}$$

Since $\widetilde{U}$ minimizes the GP loss as is shown in the previous subsection, we conclude that $\widetilde{U}$ minimizes the loss in equation (7).

C.3 Force projection loss

In this subsection, we study the force projection (P-For) loss for approximating the projection of $\boldsymbol{F}_{\boldsymbol{z}}$ onto the $\boldsymbol{z}$-space.

Denote by

$$\widetilde{\boldsymbol{F}}(\boldsymbol{z}) := \int_\Sigma \boldsymbol{F}_{\boldsymbol{z}}(\boldsymbol{y},\boldsymbol{z})\, \mathrm{d}\pi(\boldsymbol{y}|\boldsymbol{z}) \tag{12}$$

the projected force defined using the conditional distribution

$$\mathrm{d}\pi(\boldsymbol{y}|\boldsymbol{z}) = p_{\text{ss}}(\boldsymbol{y},\boldsymbol{z})/\widetilde{p}_{\text{ss}}(\boldsymbol{z})\, \mathrm{d}\boldsymbol{y}. \tag{13}$$

We can learn $\widetilde{\boldsymbol{F}}(\boldsymbol{z})$ via the following force projection loss

$$\mathrm{L}_{\text{P-For}}(\widetilde{\boldsymbol{G}}) = \int_\Omega \left|\boldsymbol{F}_{\boldsymbol{z}}(\boldsymbol{y},\boldsymbol{z}) - \widetilde{\boldsymbol{G}}(\boldsymbol{z})\right|^2 \mathrm{d}\pi(\boldsymbol{y},\boldsymbol{z}). \tag{14}$$

To justify equation (14), we note that

$$\begin{aligned}
&\int_\Omega \left|\boldsymbol{F_z}(\boldsymbol{y},\boldsymbol{z}) - \widetilde{\boldsymbol{G}}(\boldsymbol{z})\right|^2 \mathrm{d}\pi(\boldsymbol{y},\boldsymbol{z}) \\
=&\int_\Omega \left(|\boldsymbol{F_z}(\boldsymbol{y},\boldsymbol{z})|^2 + |\widetilde{\boldsymbol{G}}(\boldsymbol{z})|^2\right) \mathrm{d}\pi(\boldsymbol{y},\boldsymbol{z}) - 2\int_\Omega \boldsymbol{F_z}(\boldsymbol{y},\boldsymbol{z})\cdot\widetilde{\boldsymbol{G}}(\boldsymbol{z})\,\mathrm{d}\pi(\boldsymbol{y},\boldsymbol{z}) \\
=:&P_1 - 2P_2.
\end{aligned}$$

The term P_2 can be simplified as

$$P_2 = \int_{\widetilde{\Omega}} \left[\int_\Sigma \boldsymbol{F_z}(\boldsymbol{y},\boldsymbol{z})\mathrm{d}\pi(\boldsymbol{y}|\boldsymbol{z})\right]\cdot\widetilde{\boldsymbol{G}}(\boldsymbol{z})\,\widetilde{p}_{\mathrm{ss}}(\boldsymbol{z})\,\mathrm{d}\boldsymbol{z} = \int_{\widetilde{\Omega}} \widetilde{\boldsymbol{F}}(\boldsymbol{z})\cdot\widetilde{\boldsymbol{G}}(\boldsymbol{z})\,\widetilde{p}_{\mathrm{ss}}(\boldsymbol{z})\,\mathrm{d}\boldsymbol{z}.$$

Therefore, we have the equivalence

$$\min \mathrm{L}_{\text{P-For}}(\widetilde{\boldsymbol{G}}) \quad\Longleftrightarrow\quad \min \widetilde{\mathrm{L}}_{\text{P-For}}(\widetilde{\boldsymbol{G}}), \tag{15}$$

where

$$\widetilde{\mathrm{L}}_{\text{P-For}}(\widetilde{\boldsymbol{G}}) := \int_{\widetilde{\Omega}} \left|\widetilde{\boldsymbol{F}}(\boldsymbol{z}) - \widetilde{\boldsymbol{G}}(\boldsymbol{z})\right|^2 \widetilde{p}_{\mathrm{ss}}(\boldsymbol{z})\,\mathrm{d}\boldsymbol{z}.$$

From the analysis above we can conclude that $\widetilde{F}(\boldsymbol{z})$ minimizes the loss in equation (14).

C.4 HJB equation for the reduced potential

In this subsection, we show that the reduced potential $\widetilde{U}$ satisfies the projected HJB equation

$$\widetilde{\boldsymbol{F}}\cdot\nabla_{\boldsymbol{z}}\widetilde{U} + |\nabla_{\boldsymbol{z}}\widetilde{U}|^2 - D\Delta_{\boldsymbol{z}}\widetilde{U} - D\nabla_{\boldsymbol{z}}\cdot\widetilde{\boldsymbol{F}} = 0\,, \tag{16}$$

with asymptotic BC $\widetilde{U}\to\infty$ as $|\boldsymbol{z}|\to\infty$, or the reflecting BC $(\widetilde{\boldsymbol{F}}+\nabla_{\boldsymbol{z}}\widetilde{U})\cdot\widetilde{\boldsymbol{n}} = 0$ on $\partial\widetilde{\Omega}$, where $\widetilde{\boldsymbol{n}}$ denotes the unit outer normal on $\partial\widetilde{\Omega}$. We will only consider the rectangular domain case here. The argument for the unbounded case is similar.

Recall that $p_{\mathrm{ss}}(\boldsymbol{x})$ satisfies the FPE

$$\nabla\cdot(p_{\mathrm{ss}}\boldsymbol{F}) - D\Delta p_{\mathrm{ss}} = 0. \tag{17}$$

Integrating both sides of equation (17) on Σ with respect to $\boldsymbol{y}$ and utilizing the boundary condition $\boldsymbol{J}_{\mathrm{ss}}\cdot\boldsymbol{n} = 0$, where $\boldsymbol{J}_{\mathrm{ss}} = p_{\mathrm{ss}}\boldsymbol{F} - D\nabla p_{\mathrm{ss}}$, we get

$$\nabla_{\boldsymbol{z}}\cdot\left(\int_\Sigma \boldsymbol{F_z} p_{\mathrm{ss}}\,\mathrm{d}\boldsymbol{y}\right) - D\Delta_{\boldsymbol{z}}\widetilde{p}_{\mathrm{ss}} = 0. \tag{18}$$

Taking equation (12) and equation (13) into account, we obtain

$$\nabla_{\boldsymbol{z}}\cdot\left(\widetilde{p}_{\mathrm{ss}}\widetilde{\boldsymbol{F}}\right) - D\Delta_{\boldsymbol{z}}\widetilde{p}_{\mathrm{ss}} = \nabla_{\boldsymbol{z}}\cdot\widetilde{\boldsymbol{J}} = 0, \tag{19}$$

i.e., a FPE for $\widetilde{p}_{\mathrm{ss}}(\boldsymbol{z})$ with the reduced force field $\widetilde{\boldsymbol{F}}$, where $\widetilde{\boldsymbol{J}} := \widetilde{p}_{\mathrm{ss}}\widetilde{\boldsymbol{F}} - D\nabla_{\boldsymbol{z}}\widetilde{p}_{\mathrm{ss}}$. The corresponding boundary condition can be also derived by integrating the original BC $\boldsymbol{J}_{\mathrm{ss}} \cdot \boldsymbol{n} = 0$ on Σ with respect to $\boldsymbol{y}$ for $\boldsymbol{z} \in \partial\widetilde{\Omega}$, which gives

$$\widetilde{\boldsymbol{J}} \cdot \widetilde{\boldsymbol{n}} = \left(\widetilde{p}_{\mathrm{ss}}\widetilde{\boldsymbol{F}} - D\nabla_{\boldsymbol{z}}\widetilde{p}_{\mathrm{ss}}\right) \cdot \widetilde{\boldsymbol{n}} = 0. \tag{20}$$

Substituting the relation $\widetilde{p}_{\mathrm{ss}}(\boldsymbol{z}) = \exp\Big(-\widetilde{U}(\boldsymbol{z})/D\Big)$ into equation (19) and equation (20), we get equation (16) and the corresponding reflecting BC after some algebraic manipulations.

D State-dependent diffusion coefficients

In this section, we study the EPR loss for NESS systems with a state-dependent diffusion coefficient.

Consider the Itô SDE

$$\frac{\mathrm{d}\boldsymbol{x}(t)}{\mathrm{d}t} = \boldsymbol{F}(\boldsymbol{x}(t)) + \sqrt{2D}\sigma(\boldsymbol{x}(t))\dot{\boldsymbol{w}} \tag{21}$$

with the state-dependent diffusion matrix $\sigma(\boldsymbol{x})$. Under the same assumptions as in MT Section 1.1, we have the FPE

$$\nabla \cdot (p_{\mathrm{ss}}\boldsymbol{F}) - D\nabla^2 : (p_{\mathrm{ss}}a) = 0. \tag{22}$$

We show that the high dimensional landscape function U of equation (21) minimizes the EPR loss

$$\mathrm{L}_{\text{V-EPR}}(V) = \int_\Omega |\boldsymbol{F}^v(\boldsymbol{x}) + a(\boldsymbol{x})\nabla V(\boldsymbol{x})|^2_{a^{-1}(\boldsymbol{x})}\,\mathrm{d}\pi(\boldsymbol{x}), \tag{23}$$

where $\boldsymbol{F}^v(\boldsymbol{x}) := \boldsymbol{F}(\boldsymbol{x}) - D\nabla \cdot a(\boldsymbol{x})$ and $|\boldsymbol{u}|^2_{a^{-1}(\boldsymbol{x})} := \boldsymbol{u}^\top a^{-1}(\boldsymbol{x})\boldsymbol{u}$ for $\boldsymbol{u} \in \mathbb{R}^d$.

To justify equation (23), we first note that equation (22) can be rewritten as

$$\nabla \cdot (p_{\mathrm{ss}}\boldsymbol{F}^v - Da\nabla p_{\mathrm{ss}}) = 0\,, \tag{24}$$

which, together with the BC, implies the orthogonality relation

$$\int_\Omega \left(\boldsymbol{F}^v + a\nabla U\right) \cdot \nabla W\,\mathrm{d}\pi = 0 \tag{25}$$

for a suitable test function $W(\boldsymbol{x})$. Following the same reasoning used in establishing equation (10) and utilizing equation (25), we have

$$\int_\Omega |\boldsymbol{F}^v + a\nabla V|^2_{a^{-1}}\,\mathrm{d}\pi$$

$$
\begin{aligned}
&= \int_{\Omega} \left| \boldsymbol{F}^{v} + a\nabla U + a\nabla (V-U) \right|_{a^{-1}}^{2} \mathrm{d}\pi \\
&= \int_{\Omega} \left| \boldsymbol{F}^{v} + a\nabla U \right|_{a^{-1}}^{2} \mathrm{d}\pi + \int_{\Omega} \left| a\nabla (V-U) \right|_{a^{-1}}^{2} \mathrm{d}\pi.
\end{aligned}
$$

The last expression implies that $U(\boldsymbol{x})$ is the unique minimizer of $\mathrm{L}_{\text{V-EPR}}(V)$ up to a constant.

The above derivation for the state-dependent diffusion case will permit us to construct the landscape for the chemical Langevin dynamics, which will be studied in future works.

PART 2: COMPUTATION

Now we present the computational details omitted in the MT. We will first demonstrate the motivation for enhanced EPR, and then provide the training details and problem settings for landscape construction with original coordinates and dimension reduction. As mentioned in MT, we refer to the enhanced loss as

$$\mathrm{L}_{\mathrm{enh}} = \lambda_1 \, \mathrm{L}_{\mathrm{EPR}} + \lambda_2 \, \mathrm{L}_{\mathrm{HJB}} \, . \tag{26}$$

E Motivation for enhanced EPR

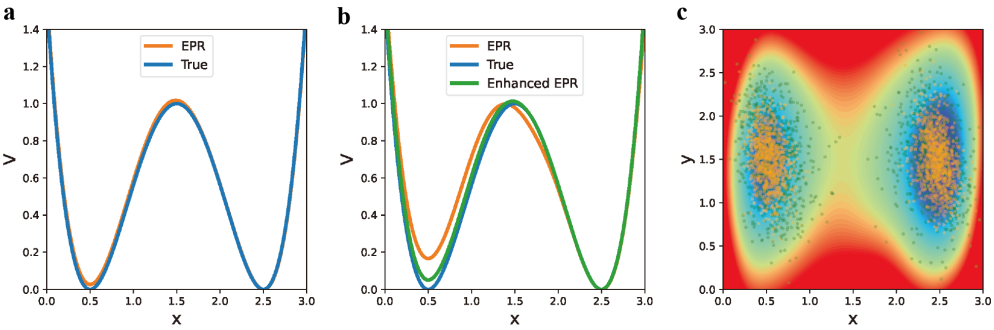

Fig. 1 An illustration for the motivation of enhanced EPR. **(a)** and **(b)** show the comparisons of the learned potentials and true solution on the line $y = 1.5$ in the toy model with $D = 0.1$ and $D = 0.05$, respectively. **(c)** shows the filled contour plot of the potential learned by only the EPR loss. The orange points are samples from the simulated invariant distribution with $D = 0.05$, while green points are enhanced samples simulated from a more diffusive distribution with $D' = 0.1$, which are used in the enhanced EPR.

We utilize enhanced samples to achieve a better coverage of the transition domain. Note that the EPR loss equation (3) in MT requires training data sampled according to $\pi(\boldsymbol{x})$. The accuracy of the learned $V(\boldsymbol{x}; \theta^*)$ (more precisely, the accuracy of the gradient $\nabla V(\boldsymbol{x}; \theta^*)$) using equation (3) in MT is guaranteed only in the "visible" domain of π, i.e., regions that are covered by sample points. However, the enhanced EPR framework equation (26) combines both the EPR loss and the HJB, which allows the use of sample data that better covers the domain Ω (e.g., more samples in the transition regions between meta-stable states and near the boundaries of the visible domain). This approach is particularly compelling when the diffusion coefficient D is relatively small.

The single EPR loss works well for the toy model with a relatively large diffusion coefficient $D = 0.1$. A slice plot of the potential at $y = 1.5$ in Fig. **1a** shows that the learned solution with EPR loss coincides well with the analytical solution. The relative root mean square error (rRMSE) and the relative mean absolute error (rMAE) have the mean and the standard deviation of 0.084 ± 0.006 and 0.066 ± 0.008 over 3 runs, respectively.

However, when decreasing D to 0.05, the samples from the simulated invariant distribution mainly stay in the double wells (orange points in Fig. **1c**) and are away from the transition region between the two wells. For this reason, as shown in Fig. **1b**, the result with a single EPR loss captures the profile of the two wells, but these two parts of the profile are not accurately connected in the transition region where there are few samples, making the left well a bit higher than the right one. We then generate enhanced samples $(\boldsymbol{x}'_i)_{1\leq i\leq N'}$ using $D' = 0.1$, which have better coverage of the transition region (green points in Fig. **1a**). Incorporating these enhanced samples, we learn the potential with the enhanced EPR loss and the result agrees better with the true solution (Fig. **1b**).

Though the enhanced EPR is more recommended in general situations, we remark that the single EPR loss can achieve competitive performance as long as the samples cover the domain effectively. This numerical experience works for all of the considered models.

F Landscape with original coordinates

In this section, we will describe the additional setup where we utilize enhanced EPR for landscape construction with original coordinates in MT. This section includes a 2D toy model, a 2D biological system with a limit cycle [4], a 2D multi-stable system [5] and a 12D Gaussian mixture model (GMM).

F.1 Training details and additional results for benchmark problems

We begin by simulating the SDE using the Euler-Maruyama scheme with reflecting boundary conditions until time $\mathsf{T} = 1000$, starting from 10000 different initial states. This simulation yields $N = 10000$ final states, which are used as the training dataset $(\mathbf{x}_i)_{1\leq i\leq N}$ to approximate the invariant distribution for EPR. For the 2D toy problem and 2D multi-stable problem, we employ a time-step size of 0.1, whereas for the 2D limit cycle problem, we use a smaller time-step size of 0.01. We train the network with a batch size of 1024 and a learning rate of 0.001 using the Adam [6] optimizer for 3000 epochs in the toy models, 5000 epochs in the multi-stable model and 10000 epochs in the limit cycle model. When focusing on single EPR loss in the toy model with $D = 0.05$, we extend to 8000 epochs to ensure adequate learning. At each training epoch, we update the dataset by performing one step Euler-Maruyama scheme to make it closer to the invariant distribution. For the comparison with normalizing flows, we train a neural spline flow [7].[1] We repeat 4 blocks of the rational quadratic spline with three layers of 64 hidden units and a followed LU linear permutation. The flow model is trained by the Adam optimizer with the learning rate 0.0001 for 20000 epochs, based on the same SDE-simulated dataset as the enhanced EPR. To ensure the robustness of our results, we perform our experiments across 5 random seeds, which helps to account for variability and establish the reliability of our findings.

[1] https://github.com/643VincentStimper/normalizing-flows

Table 1 Evaluation on various $\mu(\boldsymbol{x})$ and $\hat{\lambda}_1$ for HJB alone and enhanced EPR over 5 random seeds.

	Metrics	rRMSE				rMAE			
	$\hat{\lambda}_1$ / $\mu(\boldsymbol{x})$	**HJB** $\times$ 0.0	**Enhanced EPR** $\times$ 0.1	$\times$ 1.0	$\times$ 10.0	**HJB** $\times$ 0.0	**Enhanced EPR** $\times$ 0.1	$\times$ 1.0	$\times$ 10.0
**Toy, $D = 0.1$**	$D' = 1D$	0.034 $\pm$0.016	——	——	——	0.029 $\pm$0.014	——	——	——
	$D' = 2D$	0.168 $\pm$0.010	0.062 $\pm$0.033	**0.028** $\pm$0.010	0.038 $\pm$0.015	0.068 $\pm$0.009	0.108 $\pm$0.054	**0.026** $\pm$0.010	0.032 $\pm$0.013
	$D' = 5D$	0.225 $\pm$0.071	0.138 $\pm$0.021	0.080 $\pm$0.011	0.046 $\pm$0.018	0.123 $\pm$0.097	0.032 $\pm$0.025	0.030 $\pm$0.011	0.030 $\pm$0.019
	$D' = 10D$	0.258 $\pm$0.078	0.182 $\pm$0.033	0.095 $\pm$0.010	0.067 $\pm$0.012	0.167 $\pm$0.110	0.062 $\pm$0.040	0.038 $\pm$0.010	0.037 $\pm$0.009
**Toy, $D = 0.05$**	$D' = 1D$	0.191 $\pm$0.218	——	——	——	0.160 $\pm$0.179	——	——	——
	$D' = 2D$	0.267 $\pm$0.116	0.058 $\pm$0.045	**0.054** $\pm$0.020	0.111 $\pm$0.083	0.164 $\pm$0.120	0.049 $\pm$0.036	**0.048** $\pm$0.019	0.096 $\pm$0.069
	$D' = 5D$	0.661 $\pm$0.126	0.417 $\pm$0.162	0.204 $\pm$0.213	0.055 $\pm$0.028	0.555 $\pm$0.093	0.328 $\pm$0.155	0.127 $\pm$0.184	0.029 $\pm$0.008
	$D' = 10D$	0.544 $\pm$0.061	0.485 $\pm$0.126	0.227 $\pm$0.132	0.191 $\pm$0.190	0.489 $\pm$0.057	0.410 $\pm$0.096	0.157 $\pm$0.136	0.122 $\pm$0.168
**Multi-stable**	$D' = 1D$	0.255 $\pm$0.007	——	——	——	0.241 $\pm$0.004	——	——	——
	$D' = 2D$	0.249 $\pm$0.015	0.069 $\pm$0.045	0.110 $\pm$0.028	0.146 $\pm$0.044	0.228 $\pm$0.011	0.065 $\pm$0.046	0.106 $\pm$0.030	0.142 $\pm$0.047
	$D' = 5D$	0.628 $\pm$0.046	0.061 $\pm$0.031	0.090 $\pm$0.042	0.128 $\pm$0.043	0.553 $\pm$0.063	0.059 $\pm$0.032	0.088 $\pm$0.046	0.124 $\pm$0.044
	$D' = 10D$	0.630 $\pm$0.051	0.307 $\pm$0.036	**0.067** $\pm$0.047	0.118 $\pm$0.032	0.550 $\pm$0.066	0.197 $\pm$0.036	**0.066** $\pm$0.049	0.118 $\pm$0.034
**Limit Cycle**	$\sigma = 0.0$	0.231 $\pm$0.048	——	——	——	0.140 $\pm$0.021	——	——	——
	$\sigma = 0.05$	0.287 $\pm$0.175	0.256 $\pm$0.181	**0.070** $\pm$0.016	0.136 $\pm$0.002	0.165 $\pm$0.108	0.149 $\pm$0.107	**0.063** $\pm$0.020	0.108 $\pm$0.003
	$\sigma = 0.1$	0.503 $\pm$0.133	0.372 $\pm$0.173	0.373 $\pm$0.181	0.122 $\pm$0.008	0.313 $\pm$0.092	0.222 $\pm$0.119	0.200 $\pm$0.090	0.101 $\pm$0.006
	$\sigma = 0.2$	0.578 $\pm$0.010	0.556 $\pm$0.013	0.509 $\pm$0.025	0.298 $\pm$0.055	0.383 $\pm$0.025	0.355 $\pm$0.030	0.303 $\pm$0.040	0.175 $\pm$0.029

For a fair comparison, we fix λ_2 at 1.0 across all models. The parameters we use in the MT is $\lambda_1 = 1.0$ for toy model and multi-stable model, and $\lambda_1 = 0.01$ for the limit cycle model. As discussed in the MT, the selection of λ_1 aims to balance the two terms of the loss function. Based on our experience, systems with higher entropy production rates—typically featuring more non-gradient components in their force decomposition—require a smaller λ_1, as in the limit cycle problem. However, the specific choice of λ_1 is relatively robust. As demonstrated in Table 1, we scrutinize the stability of λ_1 by testing its sensitivity within the enhanced EPR method. We adjust λ_1 by multiplying it by a set of factors $\{$`0.0, 0.1, 1.0, 10.0`$\}$ as $\hat{\lambda}_1$ for different experiments. Then $\hat{\lambda}_1 = 0.0$ corresponds to the scenario of using HJB alone. Despite the varying distributions of $\mu(\mathbf{x})$, our selected parameters typically yield satisfactory

outcomes. Even with a relatively small $\hat{\lambda}_1$, enhanced EPR outperforms solving by HJB alone. The performance enhancements are more pronounced when relatively large values of $\hat{\lambda}_1$ are used.

We also evaluate different distributions of samples using the HJB loss, *i.e.*, $\mu(\boldsymbol{x})$. For enhanced EPR, using invariant distribution in the HJB loss term is ineffective, as HJB loss is involved to cover the transition domain. Consequently, there is no need to evaluate $\mu(\boldsymbol{x})$ with $D' = 1D$ or $\sigma = 0$ for enhanced EPR. In the case of the HJB loss, our numerical analysis suggests that overly diffusive samples can lead to significantly worse outcomes, underscoring the importance of capturing the critical domains within the loss. As shown in Table 1, the optimal results for HJB alone are achieved using the invariant distribution or a distribution slightly more extensive than that. However, with the same enhanced samples from $\mu(\boldsymbol{x})$, introducing a non-zero λ_1 enhances performance compared to HJB alone, particularly when the sample domain is so diffusive that solving HJB alone performs poorly. This emphasizes the robustness of enhanced EPR to variations in $\mu(\boldsymbol{x})$ and its advantage over solving HJB alone. Finally, we emphasize the optimal outcome for $\hat{\lambda}_1 = 1.0 \times \lambda_1$ across various $\mu(\boldsymbol{x})$ for each problem by bolding it in Table 1, serving as the result for enhanced EPR in Table 1 of the MT. While this may not be the best result among all the combinations of $\mu(\boldsymbol{x})$ and $\hat{\lambda}_1$, it is a commonly robust choice that can be readily achieved without dedicated tuning.

F.2 2D toy model

To verify the applicability and accuracy of our method, we initially apply it to a toy model with the driving force

$$\boldsymbol{F}(\boldsymbol{x}) = -(I + A)\nabla U_0(\boldsymbol{x}), \tag{27}$$

where $A \in \mathbb{R}^{d\times d}$ is a constant skew-symmetric matrix, i.e., $A^\top = -A$, and U_0 is some known function. With this choice of $\boldsymbol{F}$, one can check that the true potential landscape is simply $U(\boldsymbol{x}) = U_0(\boldsymbol{x})$. In particular, the system becomes reversible when $A = 0$. We construct a 2D toy model with the double-well potential as

$$U_0(\boldsymbol{x}) = ((x - 1.5)^2 - 1.0)^2 + 0.5(y - 1.5)^2, \tag{28}$$

where $\boldsymbol{x} = (x, y)^\top$. We take the anti-symmetric matrix

$$A = \begin{bmatrix} 0 & 0.5 \\ -0.5 & 0 \end{bmatrix}, \tag{29}$$

which introduces a counter-clockwise rotation for a focusing central force field. This sets up a simple non-equilibrium system. In this model, we have the force decomposition

$$\boldsymbol{F}(\boldsymbol{x}) = -\nabla U_0(\boldsymbol{x}) + \boldsymbol{l}(\boldsymbol{x}),\ \boldsymbol{l}(\boldsymbol{x}) = -A\nabla U_0(\boldsymbol{x})$$

and

$$\boldsymbol{l}(\boldsymbol{x}) \cdot \nabla U_0(\boldsymbol{x}) = 0, \quad \nabla \cdot \boldsymbol{l}(\boldsymbol{x}) = 0$$

hold in the pointwise sense. Therefore, the identity $\boldsymbol{l}(\boldsymbol{x}) \cdot \nabla U_0(\boldsymbol{x}) + D\nabla \cdot \boldsymbol{l}(\boldsymbol{x}) = 0$ is satisfied for any $D > 0$ and, following the discussions in MT, we have constructed a non-reversible system with analytically known double-well potential which can be used to verify the accuracy of the learned potential. We focus on the domain $\Omega = [0,3] \times [0,3]$. To fix the extra shifting degree of freedom of the potential function, we set the minimum of the potential to be zero and only plot the result on the domain $\{\boldsymbol{x}|V(\boldsymbol{x}) \leq 30D\}$ in Fig. 3 in MT since it is the domain of practical interest.

F.3 2D limit cycle model

We apply our approach to the limit cycle dynamics with a Mexican-hat shape landscape [4].

Before introducing the concrete model, let us make the following observation. For any SDE like

$$\frac{\mathrm{d}\boldsymbol{x}}{\mathrm{d}t} = \boldsymbol{F}(\boldsymbol{x}) + \sqrt{2D}\dot{\boldsymbol{w}}, \tag{30}$$

the corresponding steady FPE is

$$\nabla \cdot (p_{\mathrm{ss}}\boldsymbol{F}) - D\Delta p_{\mathrm{ss}} = 0.$$

If we make the transformation

$$\boldsymbol{F} \to \kappa\boldsymbol{F}, \ \ D \to \kappa D$$

in equation (30), then the steady state PDF

$$p_{\mathrm{ss}}(\boldsymbol{x}) = \exp\left(-\frac{U(\boldsymbol{x})}{D}\right) = \exp\left(-\frac{\kappa U(\boldsymbol{x})}{\kappa D}\right)$$

will not change. The transformation only changes the timescale of the dynamics equation (30) from t_0 to κt_0. However, the potential changes from U to κU if we utilize the drift $\kappa\boldsymbol{F}(\boldsymbol{x})$ and noise strength κD in the system equation (30). This observation allows us to set the scale of U to be $O(1)$ by adjusting κ suitably for a specific problem. An alternative approach to accomplish this task is by choosing $\boldsymbol{F}$ to be $\kappa\boldsymbol{F}$ in the EPR loss.

We take $D = 0.1$ and consider the stochastic dynamics equation (30) with $\boldsymbol{F} = (F_x, F_y)$ and

$$F_x(x,y) = \kappa\left(\frac{\alpha^2 + x^2}{1+x^2}\frac{1}{1+y} - ax\right), \tag{31}$$

$$F_y(x,y) = \frac{\kappa}{\tau_0}\left(b - \frac{y}{1+cx^2}\right), \tag{32}$$

where the parameters are $\kappa = 100, \alpha = a = b = 0.1, c = 100$, and $\tau_0 = 5$. Here the choice of $\kappa = 100$ is made such that $U \sim O(1)$ following [8]. We focus on the domain $\Omega = [0,8] \times [0,6]$. As explained in the paragraph above, our setting corresponds to the case $D = 0.1/\kappa = 0.001$ for the force field considered in [4].

F.4 2D multi-stable model

We also study the dynamics equation (30) for a multi-stable system [5] with $\boldsymbol{F} = (F_x, F_y)$ and

$$F_x(x, y) = \frac{ax^n}{S^n + x^n} + \frac{bS^n}{S^n + y^n} - k_1 x, \tag{33}$$

$$F_y(x, y) = \frac{ay^n}{S^n + y^n} + \frac{bS^n}{S^n + x^n} - k_2 y, \tag{34}$$

where the parameters are $a = b = k_1 = k_2 = 1$, $S = 0.5$, and $n = 4$. We focus on the domain $\Omega = [0, 3] \times [0, 3]$ and present the results for $D = 0.01$ in MT.

F.5 12D GMM model: high-dimensional potential

As mentioned in MT Methods 3.4, we can construct models with known potential with driving force like

$$\boldsymbol{F}(\boldsymbol{x}) = -(I + A)\nabla U_0(\boldsymbol{x}). \tag{35}$$

The true solution U_0 is denoted by $U_0(\boldsymbol{x}) = -D \log p_0(\boldsymbol{x})$, where p_0 is the probability distribution of the GMM, as defined in MT Section 1.3. We randomly generate a matrix A_0 with elements uniformly in $[0, 1]$, then obtain an skew-symmetric matrix by $A = (A_0 - A_0^\top)/2$. The non-reversible dynamics is then constructed with driving force in equation (35). We set the noise strength as $D = 0.01$ for this problem and use $D' = 5D$ for enhanced samples. We use a three-layer neural network with 80 hidden states per layer. The data size is 10000 and we use Adam with a learning rate of 0.001 and batch size of 2048. We train enhanced EPR with $\lambda_1 = 0.1, \lambda_2 = 1.0$ for 5000 epochs.

G Landscape with reduced coordinates

In this section, we provide additional details and results related to the dimension reduction problem. First, we present additional experiments of the 3D Ferrell's cell cycle problem [9]. We then demonstrate an 8D limit cycle dynamics [10], in which interesting properties emerge with projected force $\widetilde{\boldsymbol{G}}(\boldsymbol{z}; \theta^*)$ and potential $\widetilde{V}(\boldsymbol{z}; \theta^*)$. Finally, we present a 52D multi-stable dynamics [11], on which we compare the reduced potential $\widetilde{V}(\boldsymbol{z}; \theta^*)$ by two dimensionality reduction approaches.

G.1 Ferrell's three-ODE model: reduced potential

We utilize the dimensionality reduction method on Ferrell's three-ODE model for a simplified cell cycle dynamics [9], where the concentrations of the cyclin-dependent protein kinase (CDK1), Polo-like kinase 1 (Plk1), and the anaphase-promoting complex (APC), denoted by

$$x = [\text{CDK1}],\ \ y = [\text{Plk1}],\ \ z = [\text{APC}]$$

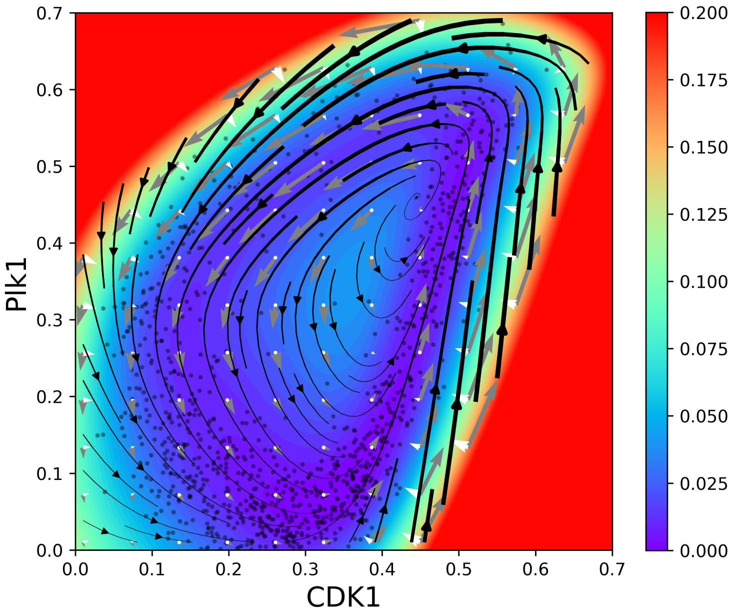

Fig. 2 Streamlines of the projected force $\widetilde{\boldsymbol{G}}(\boldsymbol{z})$ and filled contour plot of the reduced potential $\widetilde{V}(\boldsymbol{z};\theta^*)$ for Ferrell's three-ODE model learned by enhanced EPR. The projected force field $\widetilde{\boldsymbol{G}}(\boldsymbol{z})$ is decomposed into the gradient part $-\nabla\widetilde{V}(\boldsymbol{z};\theta^*)$ (white arrows) and the non-gradient part (gray arrows). The length of an arrow corresponds to the magnitude of the vector. The solid dots are samples from the simulated invariant distribution.

respectively, obey the ODEs in the domain $\Omega = [0,1]^3$

$$F_x(x,y,z) = \alpha_1 - \beta_1 x \frac{z^{n_1}}{K_1^{n_1} + z^{n_1}}, \tag{36}$$

$$F_y(x,y,z) = \alpha_2\,(1-y)\,\frac{x^{n_2}}{K_2^{n_2} + x^{n_2}} - \beta_2 y, \tag{37}$$

$$F_z(x,y,z) = \alpha_3\,(1-z)\,\frac{y^{n_3}}{K_3^{n_3} + y^{n_3}} - \beta_3 z, \tag{38}$$

with $\alpha_1 = 0.1, \alpha_2 = \alpha_3 = \beta_1 = 3, \beta_2 = \beta_3 = 1, K_1 = K_2 = K_3 = 0.5, n_1 = n_2 = 8$, and $n_3 = 8$. Since our focus in this paper is on the methodology of constructing the potential landscape, we refer the interested readers to the literature [9] for concrete biological meaning of the considered variables. We add the noise scale $D = 0.01$ with isotropic temporal Gaussian white noise. For this particular problem, we employ three-layer neural networks with 80 hidden units in each layer. We generate enhanced samples $(\mathbf{x}'_i)_{1\le i\le 10000}$ by simulating from a more diffusive distribution with $D' = 5D$. The projected force $\widetilde{\mathbf{G}}(\mathbf{z};\theta^*)$ is trained by the loss equation (14) for 1000 epochs using Adam optimizer with a learning rate 0.001 and a batch size 2048. The reduced variables $\mathbf{z} = (x,y)^\top$ are utilized during this training phase. Subsequently, we train the projected potential $\widetilde{V}(\mathbf{z};\theta^*)$ for 4000 epochs using the enhanced EPR loss defined

in equation (26), with the chosen values of $\lambda_1 = 0.1$ and $\lambda_2 = 1.0$. As shown in Fig. 2, the obtained reduced potential shows a plateau in the centering region and a local-well tube domain along the reduced limit cycle.

G.2 8D complex system: reduced potential

We consider an 8D system in which the dynamics and parameters are the same as in the supplementary information of [10]. We take CycB and Cyc20 as the reduction variable $\boldsymbol{z}$, and set the mass in this problem as $m = 0.8$.

We first point out that the noise strength $D = 0.0005$ used in [10] is not suitable here in training neural networks since this would lead to a potential of order $O(10^{-5})$. Using the idea in SI Section F.3, we amplify the original force field $\boldsymbol{F}$ in [10] by $\kappa = 1000$ times, and take $D = 0.01$ for the transformed force field. This amounts to setting $D = 10^{-5}$ for the original force field, which is even smaller than the parameter considered in [10]. We simulate the SDE without boundaries with timestep 10^{-5} until $\mathsf{T} = 5$, starting from initial states drawn from a uniform distribution in $[0, 1.25]^8$. The enhanced samples $(\boldsymbol{x}'_i)_{1\leq i\leq N'}$ are obtained by adding Gaussian perturbations with a standard deviation $\sigma = 0.05$ to the SDE-simulated dataset $(\boldsymbol{x}_i)_{1\leq i\leq N}$. We only keep the data within the biologically meaningful domain of $[0, 1.25]^8$ of size 25000 for computation.

We use three-layer networks with 80 hidden states in each layer for both force and potential. For the projected force, we train 2000 epochs to obtain $\widetilde{\boldsymbol{G}}(\boldsymbol{z}; \theta^*)$. Then we conduct the enhanced EPR equation (26) with $\lambda_1 = 0.01, \lambda_2 = 1.0$ for 10000 epochs. We use Adam with a learning rate of 0.001 and batch size of 8192.

In Fig. 3, we present a more detailed picture of the reduced dynamics for the 8D model than Fig. 5c in MT. Specifically, we further show two unstable limit cycles of the projected force field obtained by reversed time integration (two green circles in Fig. 3). These two unstable limit cycles play the role of separatrices between the neighboring stable limit sets. This picture occurs due to the fact that the landscape of the considered system is very flat in the centering region. These inner limit sets are virtual in high dimensions, but they naturally appear in the reduced dynamics on the plane. Similar features might also occur in other reduced dynamics in two dimensions.

G.3 52D multi-stable system: high-dimensional and reduced potentials

For fairness, we all use a neural network with three-layer and 20 hidden states in each layer to denote the reduced force $\widetilde{\boldsymbol{G}}(\boldsymbol{z}; \theta^*)$ and potential $\widetilde{V_1}(\boldsymbol{z}; \theta^*)$, and 80 hidden states for the 52D potential $V(\boldsymbol{x}; \theta^*)$. We use enhanced samples simulated from a more diffusive distribution with $D' = 2D$. The data size is 20000 and we use Adam with a learning rate of 0.001 and batch size of 2048, still. We use $\lambda_1 = 10.0, \lambda_2 = 1.0$ in enhanced EPR equation (26). We train the force $\widetilde{\boldsymbol{G}}(\boldsymbol{z}; \theta^*)$ for 1000 epochs and conduct $\widetilde{V_1}(\boldsymbol{z}; \theta^*)$ by enhanced EPR with for 5000 epochs. We also learn a 52D potential $V(\boldsymbol{x}; \theta^*)$ by enhanced EPR for 5000 epochs and then project it to $\widetilde{V_2}(\boldsymbol{z}; \theta^*)$ by equation (6) for 1000 epochs.

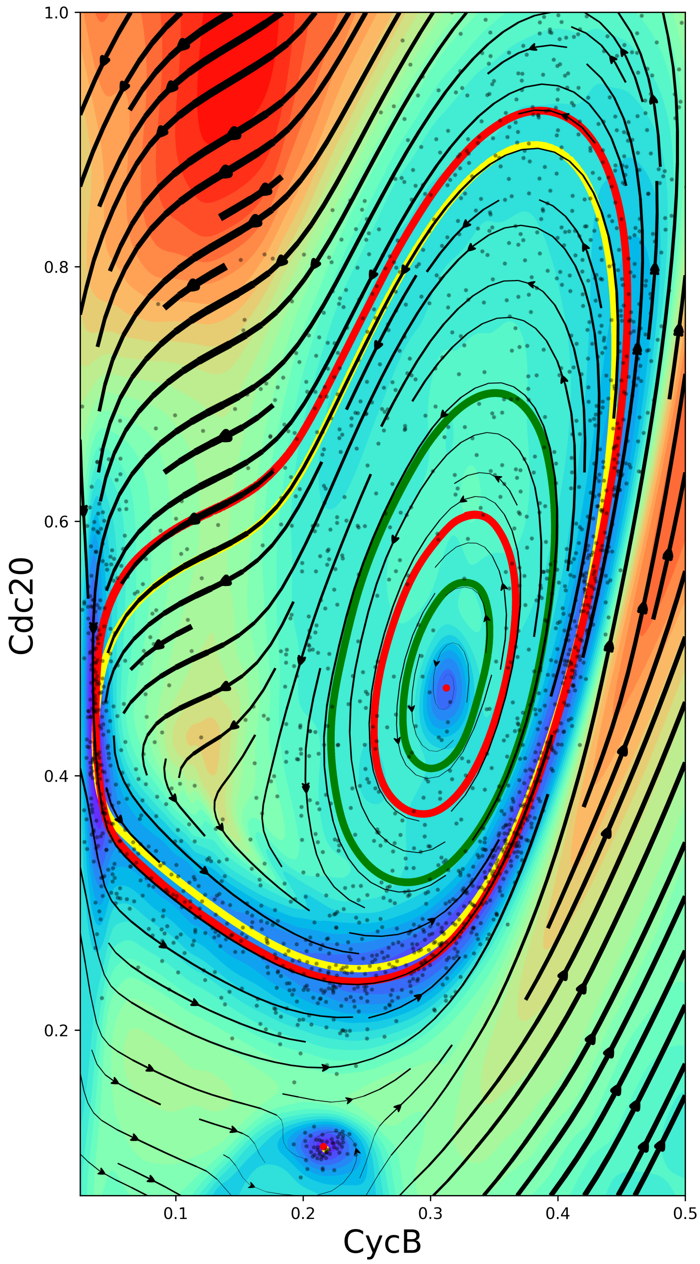

Fig. 3 Streamlines and limit sets of the projected force field of the 8D cell cycle model by two reduced variables CycB and Cdc20. The outer red circle is the stable limit cycle of the reduced force field corresponding to the yellow circle as the projection of the original high-dimensional limit cycle. The inner red circle, red dot and two green circles are stable and unstable limit sets of the reduced dynamics, respectively, which are virtual in high dimensions.

References

[1] Evans, L.C.: Partial Differential Equations, 2nd edn. American Mathematical Society, Rode Island (2010)

[2] Holmes, M.H.: Introduction to Perturbation Methods, 2nd edn. Springer, New York (2013)

[3] Frenkel, D., Smit, B.: Understanding Molecular Simulation: From Algorithms to Applications, 2nd edn. Academic Press, San Diego (2002)

[4] Wang, J., Xu, L., Wang, E.: Potential landscape and flux framework of nonequilibrium networks: Robustness, dissipation, and coherence of biochemical oscillations. Proc. Nat. Acad. Sci. USA **105**, 12271 (2008)

[5] Wang, J., Zhang, K., Xu, L., Wang, E.: Quantifying the Waddington landscape and biological paths for development and differentiation. Proc. Nat. Acad. Sci. USA **108**, 8257 (2011)

[6] Kingma, D.P., Ba, J.: Adam: a method for stochastic optimization. In: Proceedings of the International Conference on Learning Representations (2015)

[7] Durkan, C., Bekasov, A., Murray, I., Papamakarios, G.: Neural spline flows. In: Advances in Neural Information Processing Systems. NeurIPS, vol. 32 (2019)

[8] Lin, B., Li, Q., Ren, W.: Computing the invariant distribution of randomly perturbed dynamical systems using deep learning. J. Sci. Comp. **91**, 77 (2022)

[9] Ferrell, J.E., Tsai, T.Y.-C., Yang, Q.: Modeling the cell cycle: Why do certain circuits oscillate? Cell **144**(6), 874–885 (2011)

[10] Wang, J., Li, C.H., Wang, E.: Potential and flux landscapes quantify the stability and robustness of budding yeast cell cycle network. Proc. Nat. Acad. Sci. USA **107**, 8195 (2010)

[11] Li, C., Wang, J.: Quantifying cell fate decisions for differentiation and reprogramming of a human stem cell network: Landscape and biological paths. PLoS Comput. Biol. **9**, 1003165 (2013)
